# A twinning bare bones particle swarm optimization algorithm

**DOI:** 10.1371/journal.pone.0267197

**Published:** 2022-05-02

**Authors:** Jia Guo, Binghua Shi, Ke Yan, Yi Di, Jianyu Tang, Haiyang Xiao, Yuji Sato

**Affiliations:** 1 School of Information and Communication Engineering, Hubei University of Economics, Wuhan, China; 2 Smart Business Department of China Construction Third Engineering Bureau Installation Engineering Co., Ltd., Wuhan, China; 3 Faculty of Computer and Information Science, Hosei University, Tokyo, Japan; Torrens University Australia, AUSTRALIA

## Abstract

A twinning bare bones particle swarm optimization(TBBPSO) algorithm is proposed in this paper. The TBBPSO is combined by two operators, the twins grouping operator (TGO) and the merger operator (MO). The TGO aims at the reorganization of the particle swarm. Two particles will form as a twin and influence each other in subsequent iterations. In a twin, one particle is designed to do the global search while the other one is designed to do the local search. The MO aims at merging the twins and enhancing the search ability of the main group. Two operators work together to enhance the local minimum escaping ability of proposed methods. In addition, no parameter adjustment is needed in TBBPSO, which means TBBPSO can solve different types of optimization problems without previous information or parameter adjustment. In the benchmark functions test, the CEC2014 benchmark functions are used. Experimental results prove that proposed methods can present high precision results for various types of optimization problems.

## Introduction

The particle swarm optimizer (PSO) has evolved into a group of algorithms since it was first introduced by Kennedy and Eberhard in 1995. The PSOs belong to the classical evolutionary algorithm, which is inspired by the behavior of birds flocking and fish schooling. Based on the PSOs, more and more swarm evolutionary algorithms are proposed, like as artificial flora algorithm [1], artificial bee colony algorithm [2], fish swarm algorithm [3], firefly algorithm [4], cuckoo search algorithm [5]. The inspiration for these algorithms comes from the migration and reproduction processes of the swarm. In general, the PSO algorithms specialize in solving nonlinear non-stationary problems and have been widely used in parallel computing [6], pattern recognition [7], automatic control [8], transportation engineering [9] and other fields. For example, Shen [10] designs an effective gas cyclone method by using a hybrid PSO and differential evolution algorithm, and shows that the method with higher efficiency and low cost. Sung [11] proposes a method to increase the accuracy of user positioning in indoor environments using wireless-fidelity (Wi-Fi). The core point lies in the PSO algorithm and the selection of its initial weight, and the experiments have demonstrated that the method can achieve higher positioning accuracy. Li [12] presents a novel PSO-based method for hybrid wind turbine towers which is able to reduce the direct investment, labor cost, mechanical cost, and maintenance cost.

Many interesting variants of the standard PSO algorithm have been derived, like the comprehensive learning particle swarm optimizer (CLPSO) [13], the cooperative particle swarm optimizer (CPSO) [14], the hierarchical particle swarm optimizer (HPSO) [15], the unified particle swarm optimizer (UPSO) [16], and the bare bones particle swarm optimizer (BBPSO) [17]. Among them, the BBPSO algorithm as one of its typical mutated versions has also attracted a lot of attention and has been successfully applied to power systems, chemical testing, disease diagnosis, etc. For instance, Zhang [18] presents a hybrid improved BBPSO algorithm to solve dynamic economic dispatch problems. Zhang [19] proposes two evolutionary BBPSO-based feature optimization. Overall, the BBPSO algorithm is simple and easy to implement, just like other swarm algorithms, to find high-precision results for benchmark functions.

The PSO algorithm and its variants have performed relatively well in solving different types of practical problems. However, many researchers still work to overcome the defects of the PSO algorithm like the curse of dimensionality [14], the collapse of the swarm, the premature convergence, the complex multimodal problems, and so on [15]. Meanwhile, in academia and industry areas, a huge number of complex real-world problems are waiting to be solved. Therefore, the variant of PSO algorithms with superior performance is always needed.

## Related works

Researchers try to use different evolutionary method like [20–23]. By reviewing the literature, we can summarize that there are roughly three types of variants of PSO algorithms. The first type of variant focus on the initial weights. Since the original PSO algorithm did not have an inertia weight, Shi [24] proposed a modified PSO algorithm by adding an inertia weight and achieving a faster convergence. Subsequently, a constriction factor was added to the convergence behavior of the PSO algorithm [25]. Some references [15, 26] confirmed that this improved approach has effective results on the large collection problems. In the course of engineering practice, the inertia constant is typically taken to be 0.9 [27].

The second type of variant is concerned with the optimal performance of the particles. Angeline [28] proposed an entirely different approach by introducing a form of selection mechanism that allows some good particles to replace some less effective ones. The multiple neighborhoods are applied to the particle population and each neighborhood keeps its own local best solution [29]. This method is not prone to get trapped in local minima but usually has a slow convergence rate. To address this drawback, cooperative methods were introduced into distributed architectures [14]. In [30], Rui and James argued that each individual is influenced not only by the best performer among its neighbors but also by the success of all its neighbors, based on which they designed a fully informed particle swarm optimizer and confirmed its effectiveness using a benchmark function.

The third type of variant centers on making the PSO algorithm simpler. BBPSO is probably the simplest of all PSO variants and has the potential to solve single-objective unconstrained optimization problems. Details can be found in Eq 1 and Algorithm 1.

$$\begin{array}{c} \begin{array}{c} \alpha =\frac{\left(pbest\left(x_{i}^{t}\right)+Gbest^{t}\right)}{2}\\ \beta \;=|pbest\left(x_{i}^{t}\right)-Gbest^{t}|\\ x_{i}^{t+1}=GD\left(\alpha ,\beta \right) \end{array} \end{array}$$ (1)

where the $pbest(x_{i}^{t})$ is the personal best position of the particle *i* in (*t*)th generation, *Gbest*^*t*^ is the personal best position of the global best particle in (*t*)th generation, $x_{i}^{t+1}$ is the candidate new position for particle *i* in (*t* + 1)th generation, *GD*(*α*, *β*) is the Gaussian distribution with a mean *α* and a standard deviation *β*.

**Algorithm 1** BBPSO

**Require**: Max iteration time, *T*

**Require**: Fitness function, *F*

**Require**: Search Space, *R*

**Require**: Particle swarm *X* = *x*_1_, *x*_2_, … *x*_*n*_

1: Randomly generate the initial position of *X*

2: Calculate the *Pbest*_*value*, personal best value of particles

3: the *Pbest*_*position*, personal best position of particles

4: Record the *Gbest*_*position*, global best position of the swarm

5: Record the *Gbest*_*value*, global best value of the swarm

6: *t* = 0, *t* stands for the iteration times

7: **while** *t* < *T* **do**

8:  *t* = *t* + 1

9:  **for** *i* in range (1, *n*) **do**

10:   Chose a new position for *x*_*i*_ by Eq (1)

11:  **end for**

12:  Update *Pbest*_*value*

13:  Update *Pbest*_*position*

14:  Update *Gbest*_*value*

15:  Update *Gbest*_*position*

16: **end while**

17: Output *Gbest*_*value*

18: Output *Gbest*_*position*

However, it has the disadvantage of being close to collapse. In [31], Tim proved that a collapse-free condition can be obtained by including the motion of the informant, i.e., allowing a small random search over the entire search space at any stage of the optimization. Zhang [18] designed an adaptive interference factor and a new genetic operator was incorporated into the improved BBPSO. Experimental results showed that the improved method has enhanced its searchability. Mauroas [32] proposed a new method that the positions of the particles are chosen from a multivariate t-distribution and obey the rules adapted to their scale matrix. In [33], Li proposed a new BBPSO-based method, in which the behavior of the particles should obey the principle of the first-order difference equation.

Our team has been focused on improving BBPSO. In the beginning, we design BBPSO-based method that two particle work in pair [34]. In this algorithm, two particles form as a pair and are placed in different sets of evolutionary strategies. Then, we combined a local search strategy with the BBPSO in [35]. In the same way, we use a dynamic allocation strategy to enhance the search ability of BBPSO in [36]. In recent research work, we have adopted a fission-fusion strategy aimed at partitioning the search space [37]. Base on it, we proposed a fission-fusion hybrid bare-bone particle swarm optimizer (FHBBPSO) algorithm, which combines the fission strategy and the fusion strategy, and the particles are assigned to different local groups to sample the corresponding regions.

## Proposal of the twinning bare bones particle swarm optimization algorithm

In this paper, only minimum problems will be discussed, hence a *better* position in this paper stands for a position with a smaller fitness value. When several positions are discussed, a position with the smallest fitness value is defined as the *best* position. The particle with a better position is defined as a *better* particle.

The twinning bare bones particle swarm optimization algorithm (TBBPSO) is proposed in this section. In TBBPSO, two particles will form a twin and perform collaborative computing across iterations. The TBBPSO is combined by two main operators, a twins grouping operator (TGO) and a merger operator (MO). The TGO aims at dividing the particle swarm into several sub-groups and the merger operator aims at merging the sub-groups. Particles search around the global best particle and their team leaders in different generations.

### The twins grouping operator

The TGO is used to divide the particle swarm into several twins. Each twin contains two particles. Inside a twin, the particle with a smaller fitness value will be pointed as the main particle, and the other one will be the side particle. The next position of a main particle is selected by Eq 2.

$$\begin{array}{c} \begin{array}{c} \gamma =\frac{\left(pbest\left(main^{t}\right)+Gbest^{t}\right)}{2}\\ \delta \;=|pbest\left(main^{t}\right)-Gbest^{t}|\\ pbest\left(main^{t+1}\right)=\{\begin{array}{c} GD\left(\gamma ,\delta \right),\;if(F\left(GD\left(\gamma ,\delta \right)\right)<F\left(pbest\left(main^{t}\right)\right))\\ pbest(main^{t})\;\;\;\;\;\;\;\;\;\;\;else \end{array} \end{array} \end{array}$$ (2)

where the *pbest*(*main*^*t*^) is the personal best position of the main particle in (*t*)th generation, *Gbest*^*t*^ is the personal best position of the global best particle in (*t*)th generation, *pbest*(*main*^*t*+1^) is the new position for the ZZmain particle in (*t* + 1)th generation, *GD*(*γ*, *δ*) is the Gaussian distribution with a mean *γ* and a standard deviation *δ*, *F* is the target test function.

The side particle of a twin is designed to search around the main particle, hence the next position of a team member is selected by Eq 3.

$$\begin{array}{c} \begin{array}{c} \theta \;=|pbest\left(main^{t}\right)-pbest\left(side^{t}\right)|\\ \varphi =\frac{\left(pbest\left(main^{t}\right)+pbest\left(side^{t}\right)\right)}{2}\\ pbest\left(side^{t+1}\right)=\{\begin{array}{c} GD\left(\theta ,\varphi \right),\;if(F\left(GD\left(\theta ,\varphi \right)\right)<F\left(pbest\left(side^{t}\right)\right))\\ pbest(side^{t})\;\;\;\;\;\;\;\;\;\;\;\;else \end{array} \end{array} \end{array}$$ (3)

where the *pbest*(*side*^*t*^) is the personal best position of the side particle in (*t*)th generation, the *pbest*(*main*^*t*^) is the personal best position of the main particle in (*t*)th generation, *pbest*(*side*^*t*+1^) is the new position for the side particle in (*t*+ 1)th generation, *GD*(*θ*, *φ*) is the Gaussian distribution with a mean *θ* and a standard deviation *φ*, *F* is the target test function. The pseudo code of the TGO is described in Algorithm 2.

**Algorithm 2** TBBPSO-Grouping

**Require**: Fitness function, *F*

**Require**: Search Space, *R*

**Require**: Particle swarm *X* = *x*_1_, *x*_2_, …, *x*_*n*_

**Require**: Number of particles, *n*, *n* should be an even number

1: Randomly generate the initial position of *X*

2: Calculate the *Pbest*_*value*, personal best value of particles

3: Calculate the the *Pbest*_*position*, personal best position of particles

4: Record the *Gbest*_*position*, global best position of the swarm

5: Record the *Gbest*_*value*, global best value of the swarm

6: *NLG* = 0, *NLG* is short for number of local group

7: **while** *X* ≠ ⌀ **do**

8:  Take two particles *x*_*i*_ and *x*_*j*_ out of *X*

9:  *x*_*i*_ and *x*_*j*_ are defied as a twin

10:  **if** *Pbest*_*value*(*i*) < *Pbest*_*value*(*j*) **then**

11:   Chose a *NewPosition* for *x*_*i*_ by Eq 2

12:   Chose a *NewPosition* for *x*_*j*_ by Eq 3

13:   *NLG* = *NLG*+1

14:  **else**

15:   Chose a *NewPosition* for *x*_*i*_ by Eq 3

16:   Chose a *NewPosition* for *x*_*j*_ by Eq 2

17:   *NLG* = *NLG*+1

18:   **if** New positions of any particles out of *R* **then**

19:    Chose a new randon position for them in *R*

20:   **end if**

21:  **end if**

22: **end while**

23: **for** *i* = 1, *i* <= *n* **do**

24:  **if** *F*(*NewPosition*(*i*)) < *Pbest*_*value*(*i*) **then**

25:   *Pbest*_*value* = *F*(*NewPosition*(*i*))

26:   *Pbest*_*position* = *NewPosition*(*i*)

27:   **if** *F*(*NewPosition*(*i*)) < *Gbest_value*(*i*)

28:    *Gbest*_*value* = *F*(*NewPosition*(*i*))

29:    *Gbest*_*position* = *NewPosition*(*i*)

30:    *i* = *i* + 1

31:   **end if**

32:  **end if**

33: **end for**

34: Select a twin as the main local group (MLG)

35: Other twins are sub local groups (SLGs)

### Merger operator

The merger operator (MO) is proposed in this section. After grouping, particles were gathered into several local groups including one main local group (MLG) and several sub-local groups (SLGs). In each iteration, the MLS will merge one SLG until no SLG exits. During this process, particles will keep playing as the team leader and the team member. In the MLG, the best particle will be the team leader and others will be members. For all particles, leaders and teammates evolve with the same equations from TGO. The pseudo-code of the MO is given in Algorithm 3.

**Algorithm 3** TBBPSO-Merger

**Require**: Fitness function, *F*

**Require**: Search Space, *R*

**Require**: Particle swarm *X* = *x*_1_, *x*_2_, …, *x*_*n*_

**Require**: Number of local groups, *NLG* > 1

**Require**: Main-local-group, MLG

**Require**: Sub-local-group, SLG

1: **while** *NLG* ≠ 1 **do**

2:  Merge one SLG with the MLG

3:  Find the main particles in the MLG

4:  Other particles in the MLG are side particles

5:  In the MLG

6:  Chose a *NewPosition* for *x*_*main*_ by Eq 2

7:  Chose a *NewPosition* for *x*_*side*_ by Eq 3

8:  In each SLG

9:  Chose *NewPosition* for *x*_*main*_ by Eq 2

10:  Chose *NewPosition* for *x*_*side*_ by Eq 3

11:  *NLG* = *NLG* − 1

12: **end while**

13: **for** *i* = 1, *i* ≤ *n* **do**

14:  **if** *F*(*NewPosition*(*i*)) < *Pbest*_*value*(*i*) **then**

15:   *Pbest*_*value* = *F*(*NewPosition*(*i*))

16:   *Pbest*_*position* = *NewPosition*(*i*)

17:   **if** *F*(*NewPosition*(*i*)) < *Gbest*_*value*(*i*) **then**

18:    *Gbest*_*value* = *F*(*NewPosition*(*i*))

19:    *Gbest*_*position* = *NewPosition*(*i*)

20:    *i* = *i* + 1

21:   **end if**

22:  **end if**

23: **end for**

### Complete process of TBBPSO

**Fig 1:**
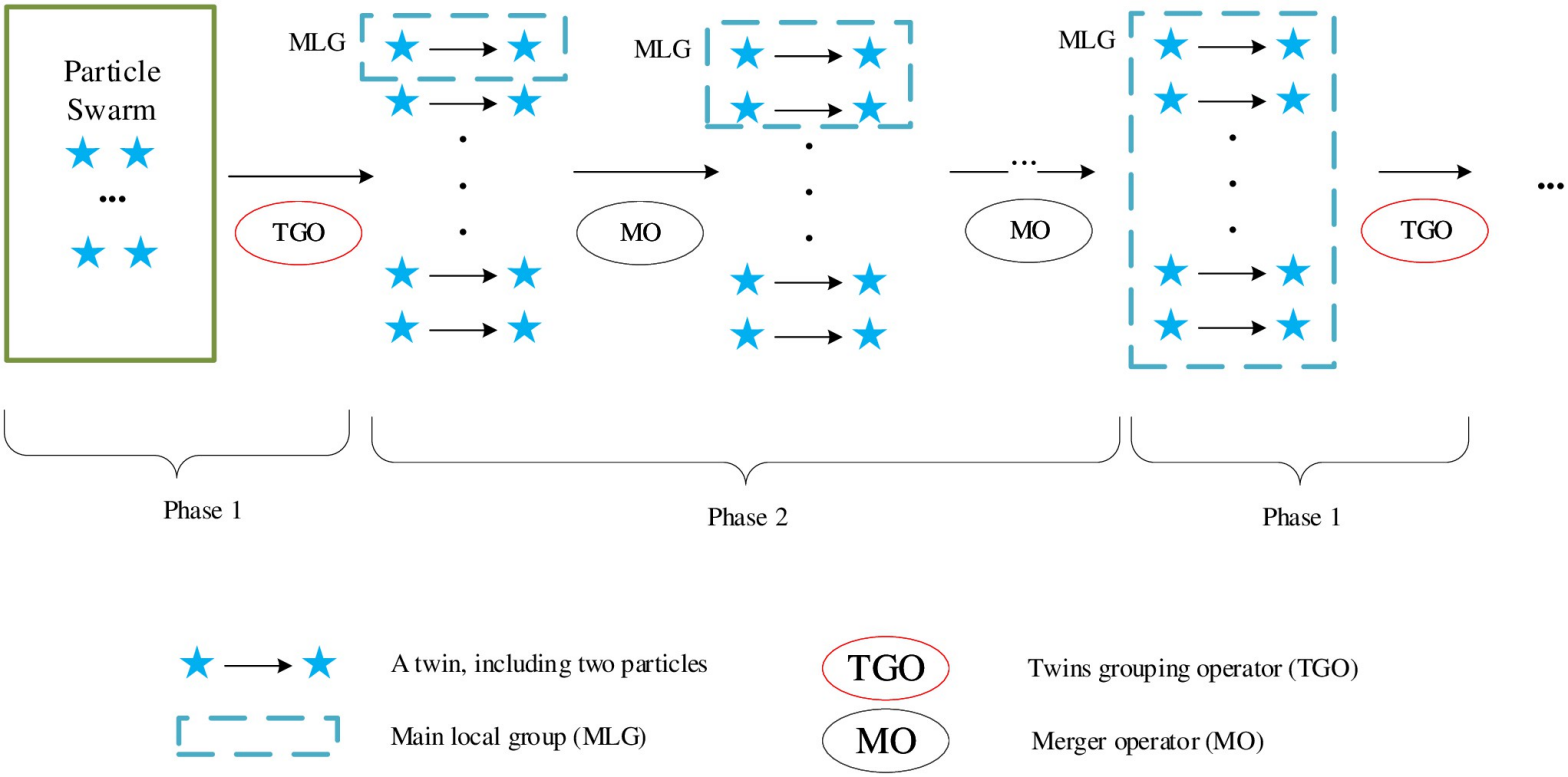
Schematic diagram of TBBPSO. Phase 1: All particles are in a same group, TGO is used to generate twins; in each twin, one particle is the group leader and the other one is the teammate; one twin will be selected as the MLG; go to Phase2. Phase 2: In each iteration, the MLG will merge one twin using MO. When all twins are in the MLG, go to Phase 1.

**Fig 2:**
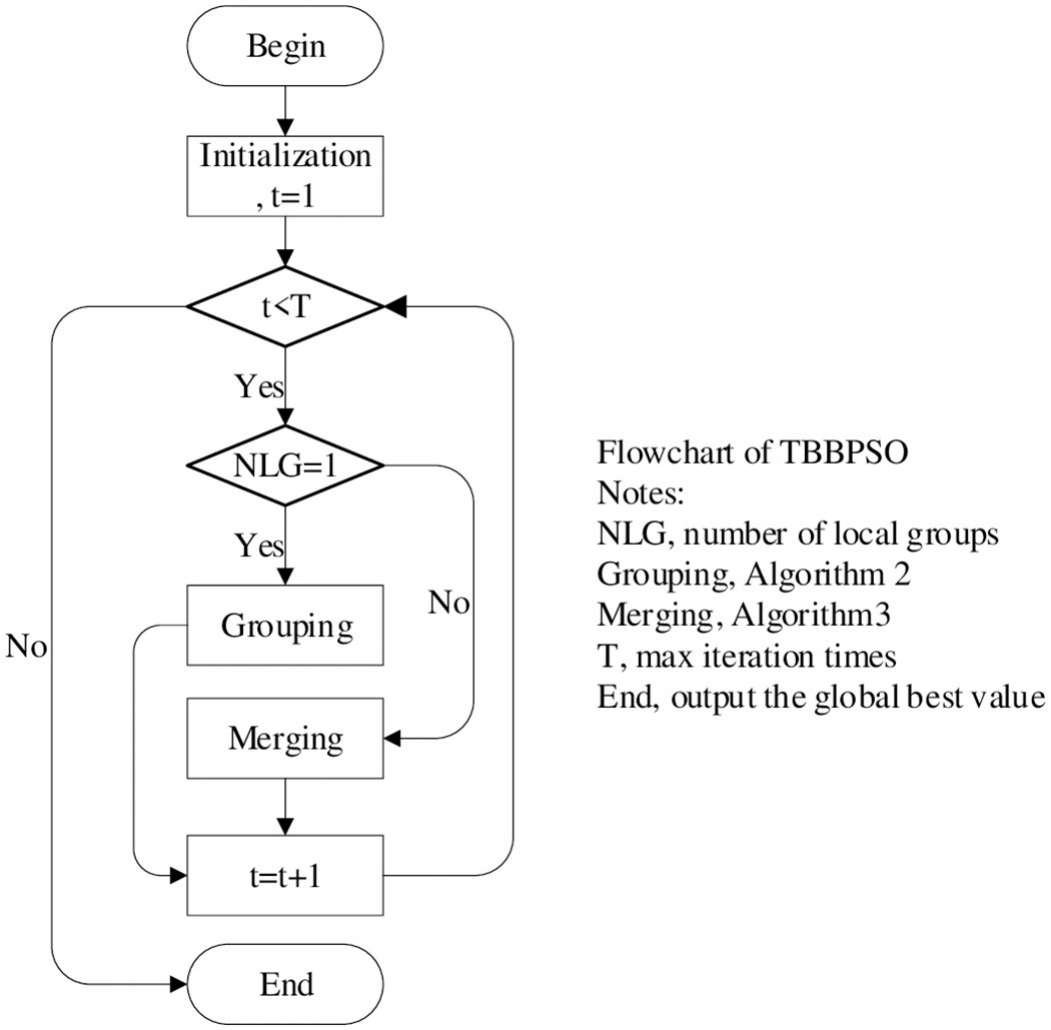
The flowchart of TBBPSO.

To describe the TBBPSO more clearly, the schematic diagram of TBBPSO is given in Fig 1, the flowchart of TBBPSO is shown in Fig 2, the pseudo-code of the TBBPSO is given in Algorithm 4.

**Algorithm 4**: TBBPSO-main

**Require**: Fitness function, *F*

**Require**: Search Space, *R*

**Require**: Particle swarm *X* = *x*_1_, *x*_2_, …, *x*_*n*_

**Require**: Number of local groups, *NLG*

**Require**: Main-local-group, *MLG*

**Require**: Sub-local-group, *SLG*

**Require**: Max iteration times, *T*

1: *t* = 1, *NLG* = 1

2: **while** *t* < *T* **do**

3:  **if** *NLG* = 1 **then**

4:   Run Algorithm 2

5:  **else**

6:   Run Algorithm 3

7:  **end if**

8:  *t* = *t* + 1

9: **end while**

10: Output *Gbest*_*value*

11: Output *Gbest*_*position*

## Experiments and results

### Experimental methods

To verify the optimization ability of the TBBPSO, the CEC 2014 benchmark functions (CEC2014BF) [38] are used in the experiments. Details of the CEC2014BF can be found in Table 1. The, BBPSO [17], PBBPSO [34] and DLS-BBPSO [35] are selected into the control group. To make a fair competition, all algorithms use the same population size, iteration times, and best parameters in their original papers. All tests are repeated 31 times and the average results are recorded to reduce accidental errors,. The population size for all algorithms is 100, dimension is 50, max generation time is 1.000E+4. The Measurement Error (ME) is defined as |*final gbest value* − *Theoretically optimal*|.

**Table 1:** Experimental functions, the CEC 2014 benchmark functions, the search range for each function is (-100,100) [38].

Types	Function	Theoretically Optimal
Unimodal Functions	*f*_1_ = Rotaten High Conditioned Elliptic Function	100
	*f*_2_ = Rotated Bent Cigar Function	200
	*f*_3_ = Rotated Discus Function	300
Simple Multimodal Functions	*f*_4_ = Shifted and Rotated Rosenbrock’s Function	400
	*f*_5_ = Shifted and Rotated ACKLEY’s Function	500
	*f*_6_ = Shifted and Rotated Weierstrass’s Function	600
	*f*_7_ = Shifted and Rotated Griewank’s Function	700
	*f*_8_ = Shifted Rastrigin’s Function	800
	*f*_9_ = Shifted and Rotated Rastrigin’s Function	900
	*f*_10_ = Shifted Schwefel’s Function	1000
	*f*_11_ = Shifted and Rotated Schwefel’s Function	1100
	*f*_12_ = Shifted and Rotated Katsure Function	1200
	*f*_13_ = Shifted and Rotated HappyCat Function	1300
	*f*_14_ = Shifted and Rotated HGBat Function	1400
	*f*_15_ = Shifted and Rotated Expanded Griewank’s plus Rosenbrock’s Function	1500
	*f*_16_ = Shifted and Rotated Expanded Scaffer’s F6 Function	1600
Hybrid Functions	*f*_17_ = Hybrid Function 1 (N = 3)	1700
	*f*_18_ = Hybrid Function 2 (N = 3)	1800
	*f*_19_ = Hybrid Function 3 (N = 4)	1900
	*f*_20_ = Hybrid Function 4 (N = 4)	2000
	*f*_21_ = Hybrid Function 5 (N = 5)	2100
	*f*_22_ = Hybrid Function 6 (N = 5)	2200
Composition Functions	*f*_23_ = Composition Function 1 (N = 5)	2300
	*f*_24_ = Composition Function 2 (N = 3)	2400
	*f*_25_ = Composition Function 3 (N = 3)	2500
	*f*_26_ = Composition Function 4 (N = 5)	2600
	*f*_27_ = Composition Function 5 (N = 5)	2700
	*f*_28_ = Composition Function 6 (N = 5)	2800
	*f*_29_ = Composition Function 7 (N = 3)	2900
	*f*_30_ = Composition Function 8 (N = 3)	3000

### Experiments with top BBPSO-based method

The FHBBPSO [37], one of the top BBPSO-based methods is selected to compare with TBBPSO. These two methods run on the Rotaten High Conditioned Elliptic Function 31 times, the population size of the two methods is 100, dimension is 50, max iteration times is 1000, and the mean MEs are shown in Table 2. It can be seen that at 100 and 200 iterations, the results of the two methods are very close. After one 1000 iterations, the result of TBBPSO is 22.16% smaller than that of FHBBPSO. It can be assumed that TBBPSO has a clear advantage in this set of experiments.

**Table 2:** MEs of TBBPSO and FHBBPSO.

Function	100 iterations	200 iterations	300 iterations	500 iterations	1000 iterations
FHBBPSO	1.208E+09	4.273E+08	2.305E+08	1.251E+08	5.5299E+07
TBBPSO	1.196E+09	3.315E+08	1.724E+08	9.117E+07	4.3047E+07

### Experimental results with complete set of benchmark functions

MEs are displayed in Tables 3 and 4. In 30 benchmark functions, TBBPSO ranked first in 12 functions and ranked second in 12 functions. In each benchmark function, the algorithm with the first rank will get 1 point, the second rank will get 2 points, the third rank will get 3 points and the fourth rank will get 4 points. The average rank point of TBBPSO is 1.900, the best of the four optimization algorithms. The Friedman statistic test [22] is also implemented and average rank results are shown in Table 4.

**Table 3:** Experimental Results, ME of BBPSO, PBBPSO, DLS-BBPSO and TBBPSO for *f*_1_–*f*_15_. Mean is the mean value from 31 independent runs, STD is the standard deviation of the 31 runs, Rank is the rank of 4 algorithms.

Function Number	Data Tpye	BBPSO [17]	PBBPSO [34]	DLS-BBPSO [35]	TBBPSO
*f*_1_	Mean	7.519E+06	7.383E+06	6.432E+06	**5.603E+06**
	STD	3.725E+06	4.026E+06	2.599E+06	**2.618E+06**
	Rank	4	3	2	**1**
*f*_2_	Mean	2.689E+04	**1.748E+04**	2.668E+04	2.532E+04
	STD	2.943E+04	**2.104E+04**	3.843E+04	2.424E+04
	Rank	4	**1**	3	2
*f*_3_	Mean	**1.420E+03**	3.148E+03	3.379E+03	2.644E+03
	STD	**1.322E+03**	2.732E+03	3.734E+03	3.464E+03
	Rank	**1**	3	4	2
*f*_4_	Mean	**5.621E+01**	5.846E+01	6.059E+01	7.049E+01
	STD	**2.434E+01**	2.435E+01	3.290E+01	3.537E+01
	Rank	**1**	2	3	4
*f*_5_	Mean	2.112E+01	2.112E+01	2.111E+01	**2.104E+01**
	STD	3.240E-02	3.240E-02	4.150E-02	**5.360E+01**
	Rank	3	4	2	**1**
*f*_6_	Mean	**3.476E+01**	5.288E+01	4.043E+01	3.813E+01
	STD	**4.688E+00**	1.600E+01	1.362E+01	6.719E+00
	Rank	**1**	4	3	2
*f*_7_	Mean	6.500E-03	1.090E-02	**3.500E-03**	6.000E-03
	STD	8.500E-03	1.260E-02	**6.500E-03**	6.800E-03
	Rank	3	4	**1**	2
*f*_8_	Mean	1.137E+02	1.017E+02	1.048E+02	**1.007E+02**
	STD	2.345E+01	2.116E+01	1.537E+01	**1.970E+01**
	Rank	4	2	3	**1**
*f*_9_	Mean	2.471E+02	2.550E+02	**2.154E+02**	2.340E+02
	STD	6.196E+01	7.284E+01	**7.018E+01**	6.264E+01
	Rank	3	4	**1**	2
*f*_10_	Mean	2.025E+03	**1.738E+03**	1.847E+03	1.962E+03
	STD	3.967E+02	**4.929E+03**	5.216E+02	4.781E+02
	Rank	4	**1**	2	3
*f*_11_	Mean	7.509E+03	1.171E+03	1.078E+04	**7.306E+03**
	STD	3.482E+03	3.953E+03	4.264E+04	**2.791E+03**
	Rank	2	4	3	**1**
*f*_12_	Mean	2.942E+00	3.181E+00	3.202E+00	**2.478E+00**
	STD	8.531E-01	2.635E-01	2.530E-01	**7.542E-01**
	Rank	2	3	4	**1**
*f*_13_	Mean	5.539E-01	5.598E-01	5.518E-01	**5.075E-01**
	STD	1.087E-01	8.210E-02	8.800E-02	**8.530E-02**
	Rank	3	4	2	**1**
*f*_14_	Mean	5.391E-01	5.597E-01	5.933E-01	**4.361E-01**
	STD	2.784E-01	2.851E-01	2.809E-01	**2.441E-01**
	Rank	2	3	4	**1**
*f*_15_	Mean	1.553E+01	1.747E+01	**1.381E+01**	1.474E+01
	STD	4.246E+00	4.542E+00	**5.512E+00**	4.344E+00
	Rank	3	4	**1**	2

**Table 4:** Experimental Results, ME of BBPSO, PBBPSO, DLS-BBPSO and TBBPSO for *f*_16_–*f*_30_. Mean is the mean value from 31 independent runs, STD is the standard deviation of the 31 runs, Rank is the rank of 4 algorithms. Average rank point is at the bottom of the table.

Function Number	Data Tpye	BBPSO [17]	PBBPSO [34]	DLS-BBPSO [35]	TBBPSO
*f*_16_	Mean	**2.074E+01**	2.179E+01	2.128E+01	2.139E+01
	STD	**8.171E-01**	1.069E+00	1.237E+00	7.566E-01
	Rank	**1**	4	2	3
*f*_17_	Mean	1.119E+06	1.058E+06	1.128E+06	**9.136E+05**
	STD	7.997E+05	6.223E+05	9.273E+05	**5.381E+05**
	Rank	3	2	4	**1**
*f*_18_	Mean	**6.771E+03**	7.806E+03	7.029E+03	7.999E+03
	STD	**6.571E+03**	1.152E+04	6.943E+03	1.145E+04
	Rank	**1**	3	2	4
*f*_19_	Mean	3.596E+01	4.385E+01	**3.401E+01**	3.584E+01
	STD	1.420E+01	2.421E+01	**1.096E+01**	1.485E+01
	Rank	3	4	**1**	2
*f*_20_	Mean	**7.135E+03**	1.926E+04	1.790E+03	1.023E+04
	STD	**6.152E+03**	1.586E+04	1.514E+04	9.197E+03
	Rank	**1**	4	3	2
*f*_21_	Mean	4.630E+05	5.050E+05	5.290E+05	**4.027E+05**
	STD	2.721E+05	4.460E+05	3.772E+05	**2.398E+05**
	Rank	2	3	4	**1**
*f*_22_	Mean	1.192E+03	1.462E+03	1.134E+03	**1.091E+03**
	STD	3.898E+02	3.851E+02	3.643E+02	**2.788E+02**
	Rank	3	4	2	**1**
*f*_23_	Mean	3.370E+02	3.370E+02	3.370E+02	3.370E+02
	STD	0.000	0.000	0.000	0.000
	Rank	1	1	1	1
*f*_24_	Mean	2.633E+02	**2.616E+02**	2.631E+02	2.647E+02
	STD	8.509E+00	**1.178E+00**	8.474E+00	0.522E+00
	Rank	3	**1**	2	4
*f*_25_	Mean	2.009E+02	2.009E+02	2.009E+02	**2.008E+02**
	STD	0.303E+00	0.275E+00	0.305E+00	**0.273E+00**
	Rank	4	2	3	**1**
*f*_26_	Mean	**1.005E+02**	1.006E+02	1.005E+02	1.005E+02
	STD	**0.101E+00**	0.071E+00	0.081E+00	0.107E+00
	Rank	**1**	4	3	2
*f*_27_	Mean	**1.269E+03**	1.892E+03	1.407E+03	1.435E+03
	STD	**1.169E+02**	3.309E+02	2.380E+02	2.206E+02
	Rank	**1**	4	2	3
*f*_28_	Mean	3.934E+02	3.934E+02	**3.867E+02**	3.889E+02
	STD	1.541E+01	1.579E+01	**1.342E+01**	1.455E+02
	Rank	3	4	**1**	2
*f*_29_	Mean	**2.246E+02**	2.295E+02	2.267E+02	2.253E+02
	STD	**2.083E+01**	2.708E+01	1.576E+01	2.032E+01
	Rank	**1**	4	3	2
*f*_30_	Mean	1.320E+03	1.275E+03	**1.203E+03**	1.246E+03
	STD	2.824E+02	3.359E+02	**2.455E+02**	3.227E+02
	Rank	4	3	**1**	2
Average Rank		2.400	3.100	2.400	**1.900**

Specifically, numerical analyses are listed below:

- In *f*_1_, TBBPSO gains the first rank, the results of TBBBOS are 12.88% better than results from DLS-BBPSO, the second-best algorithm.
- In *f*_2_, TBBPSO gains the second rank, the results of TBBBOS are 44.87% worse than results from PBBPSO, the best algorithm.
- In *f*_3_, TBBPSO gains the second rank, the results of TBBBOS are 86.22% worse than results from BBPSO, the best algorithm.
- In *f*_4_, TBBPSO gains the fourth rank, the results of TBBBOS are 25.40% worse than results from BBPSO, the best algorithm.
- In *f*_5_, TBBPSO gains the first rank, the results of TBBBOS are 0.04% better than results from DLS-BBPSO, the second-best algorithm.
- In *f*_6_, TBBPSO gains the second rank, the results of TBBBOS are 9.68% worse than results from BBPSO, the best algorithm.
- In *f*_7_, TBBPSO gains the second rank, the results of TBBBOS are 71.43% worse than results from DLS-BBPSO, the best algorithm.
- In *f*_8_, TBBPSO gains the first rank, the results of TBBBOS are 1.01% better than results from PBBPSO, the second-best algorithm.
- In *f*_9_, TBBPSO gains the second rank, the results of TBBBOS are 8.63% worse than results from DLS-BBPSO, the best algorithm.
- In *f*_10_, TBBPSO gains the third rank, the results of TBBBOS are 12.87% worse than results from PBBPSO, the best algorithm.
- In *f*_11_, TBBPSO gains the first rank, the results of TBBBOS are 2.70% better than results from BBPSO, the second-best algorithm.
- In *f*_12_, TBBPSO gains the first rank, the results of TBBBOS are 15.77% better than results from BBPSO, the second-best algorithm.
- In *f*_13_, TBBPSO gains the first rank, the results of TBBBOS are 8.03% better than results from DLS-BBPSO, the second-best algorithm.
- In *f*_14_, TBBPSO gains the first rank, the results of TBBBOS are 19.11% better than results from BBPSO, the second-best algorithm.
- In *f*_15_, TBBPSO gains the second rank, the results of TBBBOS are 0.68% worse than results from DLS-BBPSO, the second-best algorithm.
- In *f*_16_, TBBPSO gains the third rank, the results of TBBBOS are 3.13% worse than results from BBPSO, the best algorithm.
- In *f*_17_, TBBPSO gains the first rank, the results of TBBBOS are 13.63% better than results from PBBPSO, the second-best algorithm.
- In *f*_18_, TBBPSO gains the fourth rank, the results of TBBBOS are 18.13% worse than results from BBPSO, the best algorithm.
- In *f*_19_, TBBPSO gains the second rank, the results of TBBBOS are 5.39% worse than results from DLS-BBPSO, the best algorithm.
- In *f*_20_, TBBPSO gains the second rank, the results of TBBBOS are 43.42% worse than results from BBPSO, the best algorithm.
- In *f*_21_, TBBPSO gains the first rank, the results of TBBBOS are 13.02% better than results from BBPSO, the second-best algorithm.
- In *f*_22_, TBBPSO gains the first rank, the results of TBBBOS are 3.78% better than results from DLS-BBPSO, the second-best algorithm.
- In *f*_23_, four algorithms give the same results, which means 4 algorithms are trapped by the local minimal and unable to escape.
- In *f*_24_, TBBPSO gains the fourth rank, the results of TBBBOS are 1.18% worse than results from PBBPSO, the best algorithm.
- In *f*_25_, TBBPSO gains the first rank, the results of TBBBOS are 0.06% better than results from PBBPSO, the second-best algorithm.
- In *f*_26_, TBBPSO gains the second rank, the results of TBBBOS are 0.01% worse than results from BBPSO, the best algorithm.
- In *f*_27_, TBBPSO gains the third rank, the results of TBBBOS are 13.05% worse than results from BBPSO, the best algorithm.
- In *f*_28_, TBBPSO gains the second rank, the results of TBBBOS are 0.587% worse than results from DLS-BBPSO, the best algorithm.
- In *f*_29_, TBBPSO gains the second rank, the results of TBBBOS are 0.32% worse than results from BBPSO, the best algorithm.
- In *f*_30_, TBBPSO gains the second rank, the results of TBBBOS are 3.61% worse than results from DLS-BBPSO, the best algorithm.

Compared with traditional methods, TBBPSO is more reliable and more robust. The MO and the TGO work together to cross the local minimal and search for high precision results. To perform the convergence situation across iterations, the ME in different iterations for BBPSO, PBBPSO, DLS-BBPSO, and TBBPSO is shown in Figs 3–32. The scale on the vertical axis represents the value of ME. The scale on the horizontal axis represents iteration times, 100 on the horizontal axis represents 10,000 iterations.

**Fig 3:**
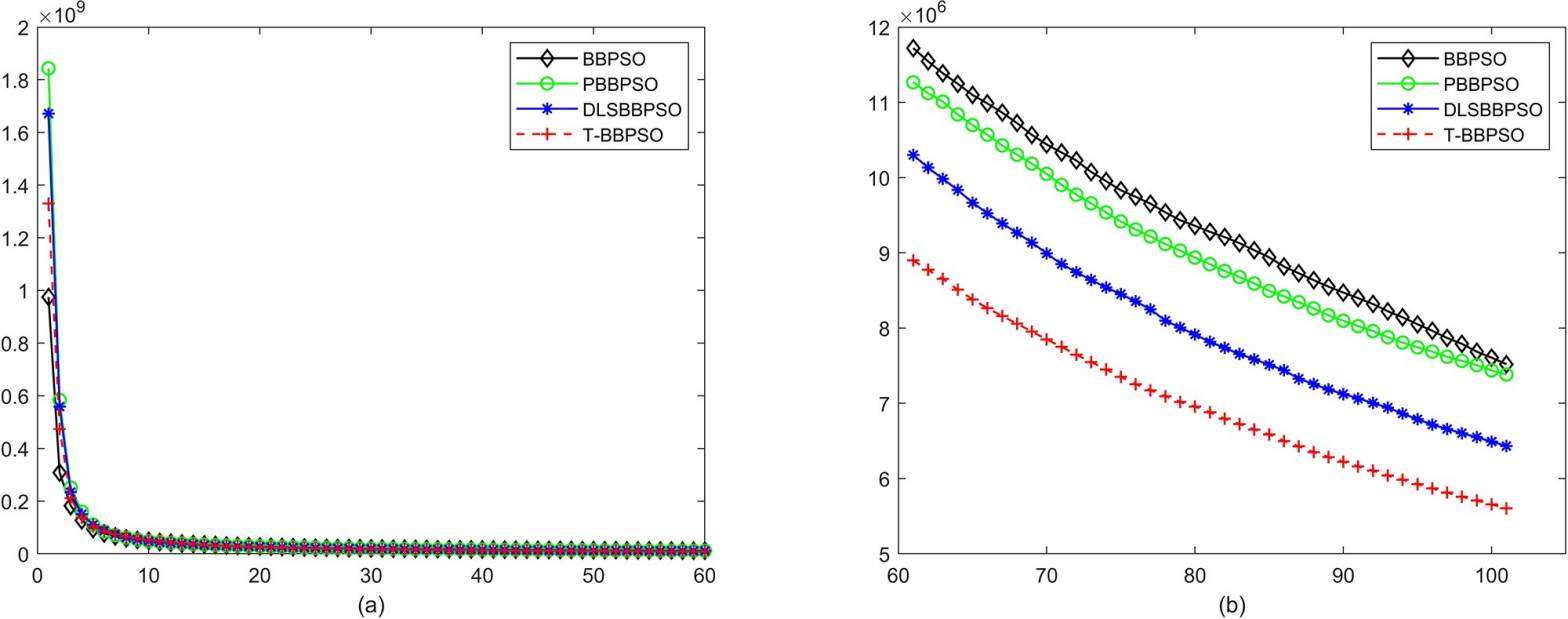
Comparison of convergence speed between BBPSO, PBBPSO, DLS-BBPSO and TBBPSO, *f*_1_, (a) iteration 0–6000, (b) iteration 6000–10000 the unit is 100 iteration.

**Fig 4:**
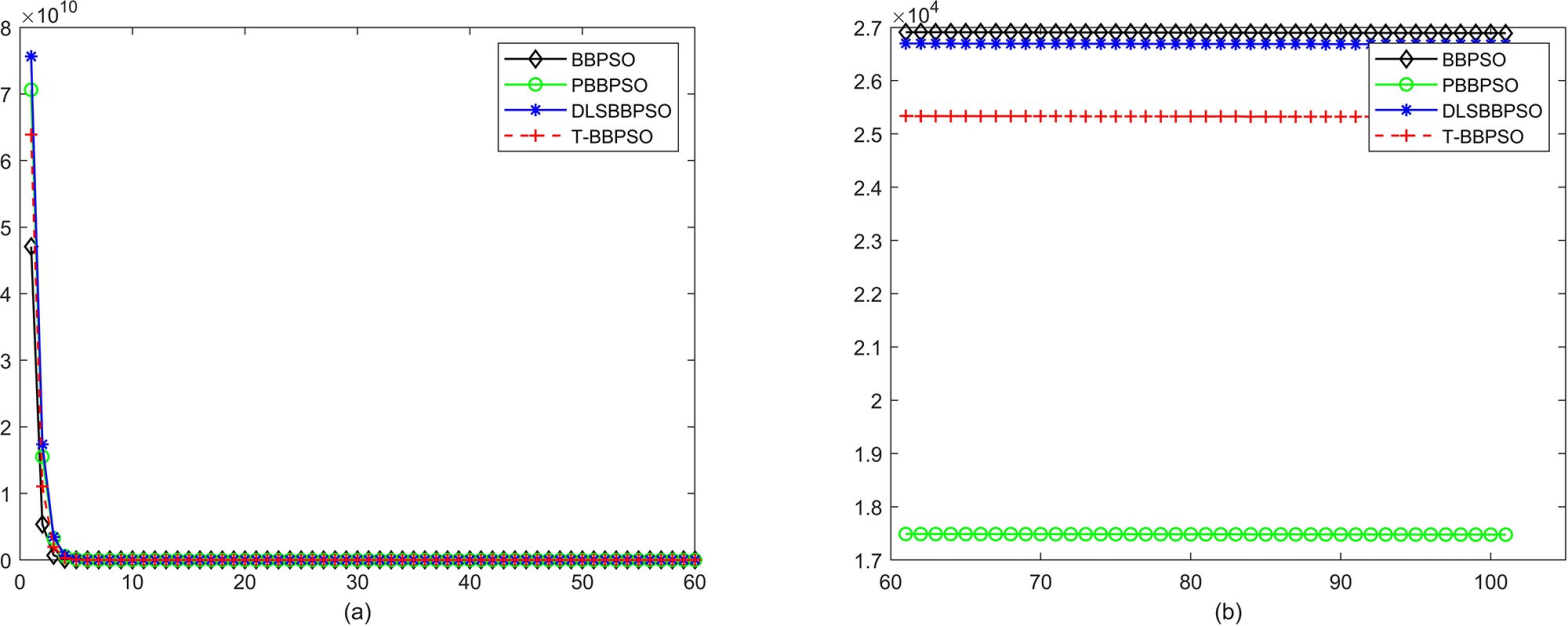
Comparison of convergence speed between BBPSO, PBBPSO, DLS-BBPSO and TBBPSO, *f*_2_, (a) iteration 0–6000, (b) iteration 6000–10000 the unit is 100 iteration.

**Fig 5:**
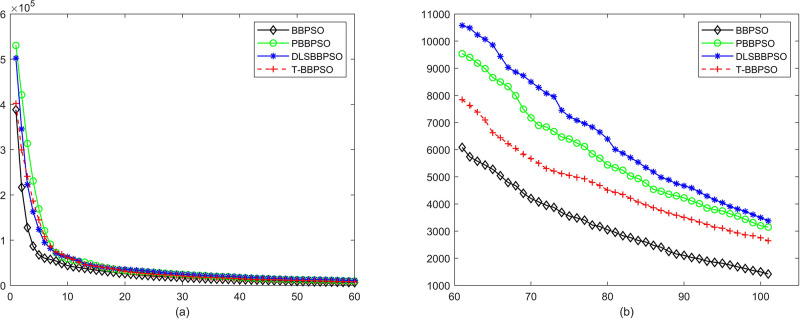
Comparison of convergence speed between BBPSO, PBBPSO, DLS-BBPSO and TBBPSO, *f*_3_, (a) iteration 0–6000, (b) iteration 6000–10000 the unit is 100 iteration.

**Fig 6:**
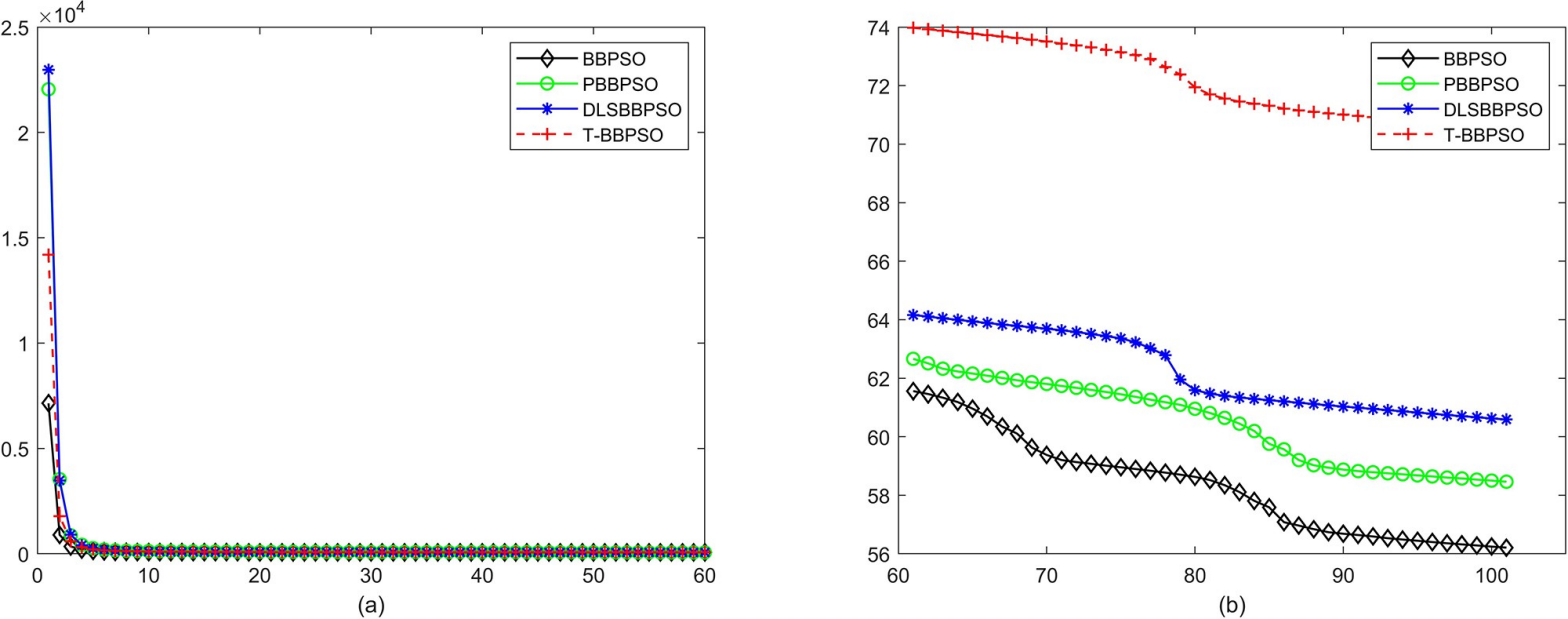
Comparison of convergence speed between BBPSO, PBBPSO, DLS-BBPSO and TBBPSO, *f*_4_, (a) iteration 0–6000, (b) iteration 6000–10000 the unit is 100 iteration.

**Fig 7:**
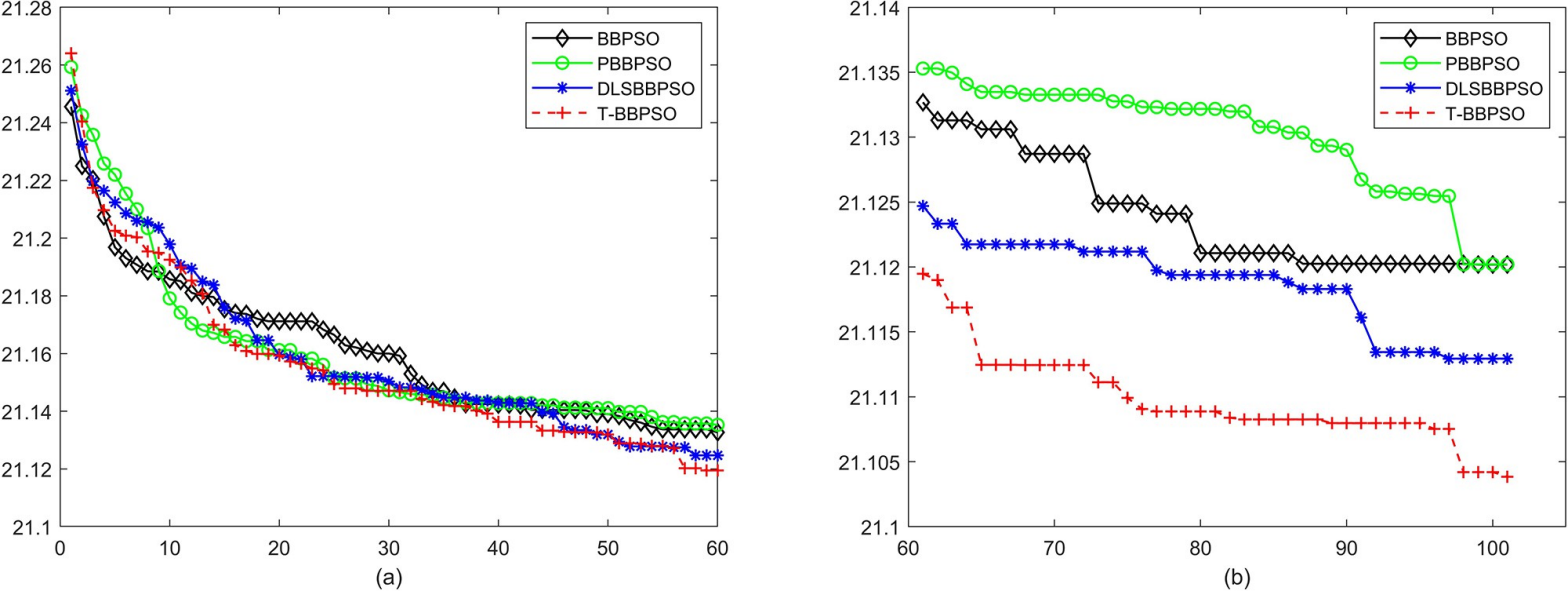
Comparison of convergence speed between BBPSO, PBBPSO, DLS-BBPSO and TBBPSO, *f*_5_, (a) iteration 0–6000, (b) iteration 6000–10000 the unit is 100 iteration.

**Fig 8:**
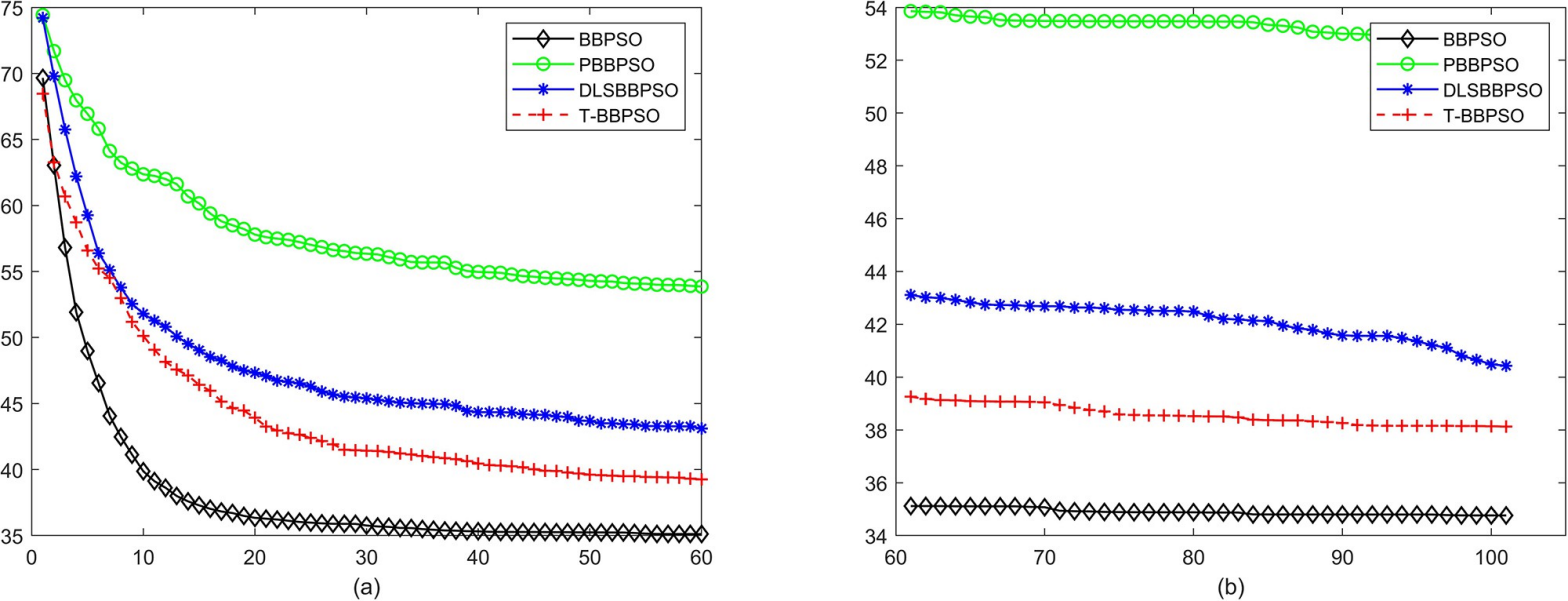
Comparison of convergence speed between BBPSO, PBBPSO, DLS-BBPSO and TBBPSO, *f*_6_, (a) iteration 0–6000, (b) iteration 6000–10000 the unit is 100 iteration.

**Fig 9:**
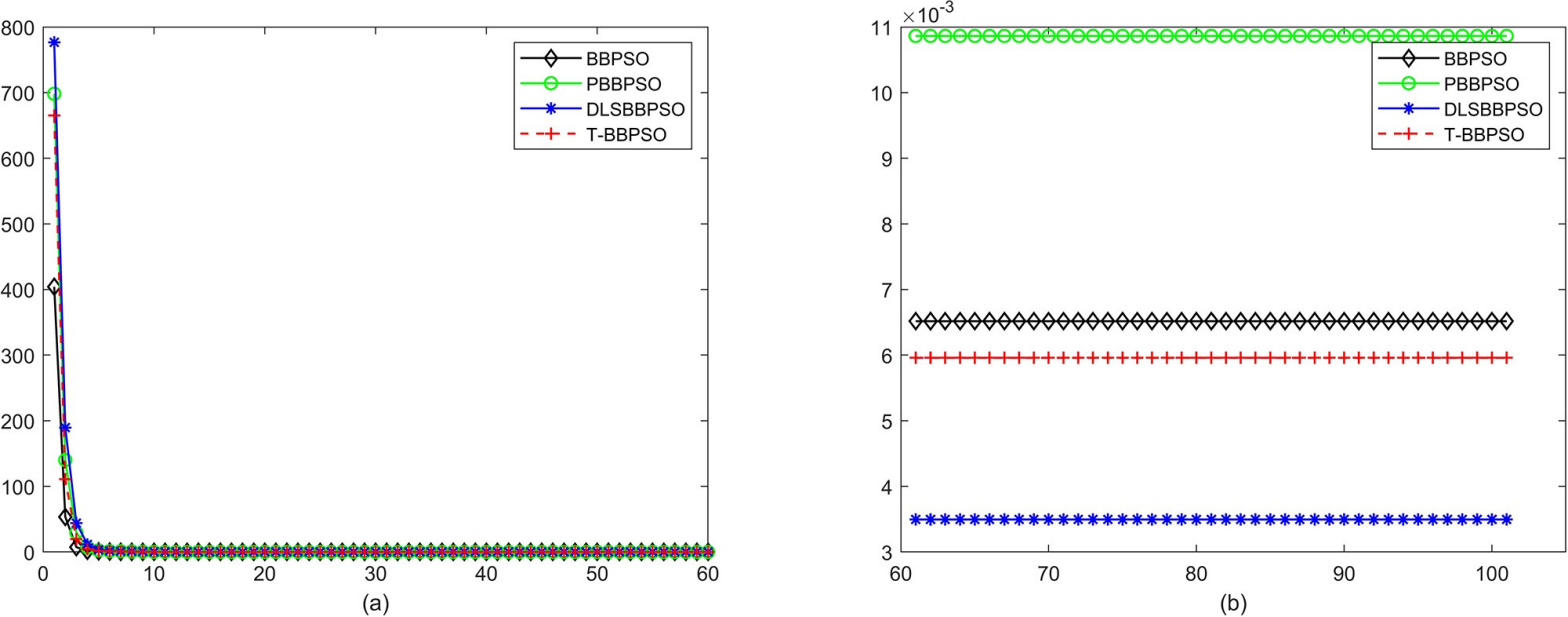
Comparison of convergence speed between BBPSO, PBBPSO, DLS-BBPSO and TBBPSO, *f*_7_, (a) iteration 0–6000, (b) iteration 6000–10000 the unit is 100 iteration.

**Fig 10:**
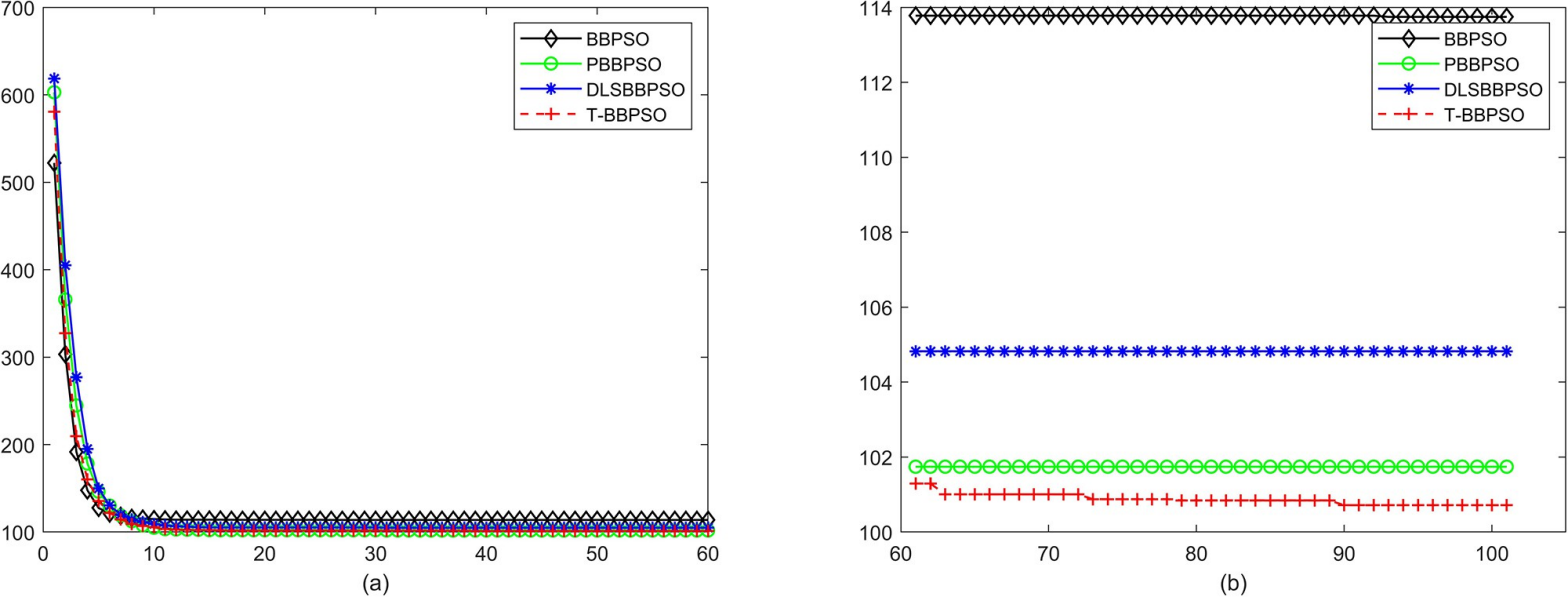
Comparison of convergence speed between BBPSO, PBBPSO, DLS-BBPSO and TBBPSO, *f*_8_, (a) iteration 0–6000, (b) iteration 6000–10000 the unit is 100 iteration.

**Fig 11:**
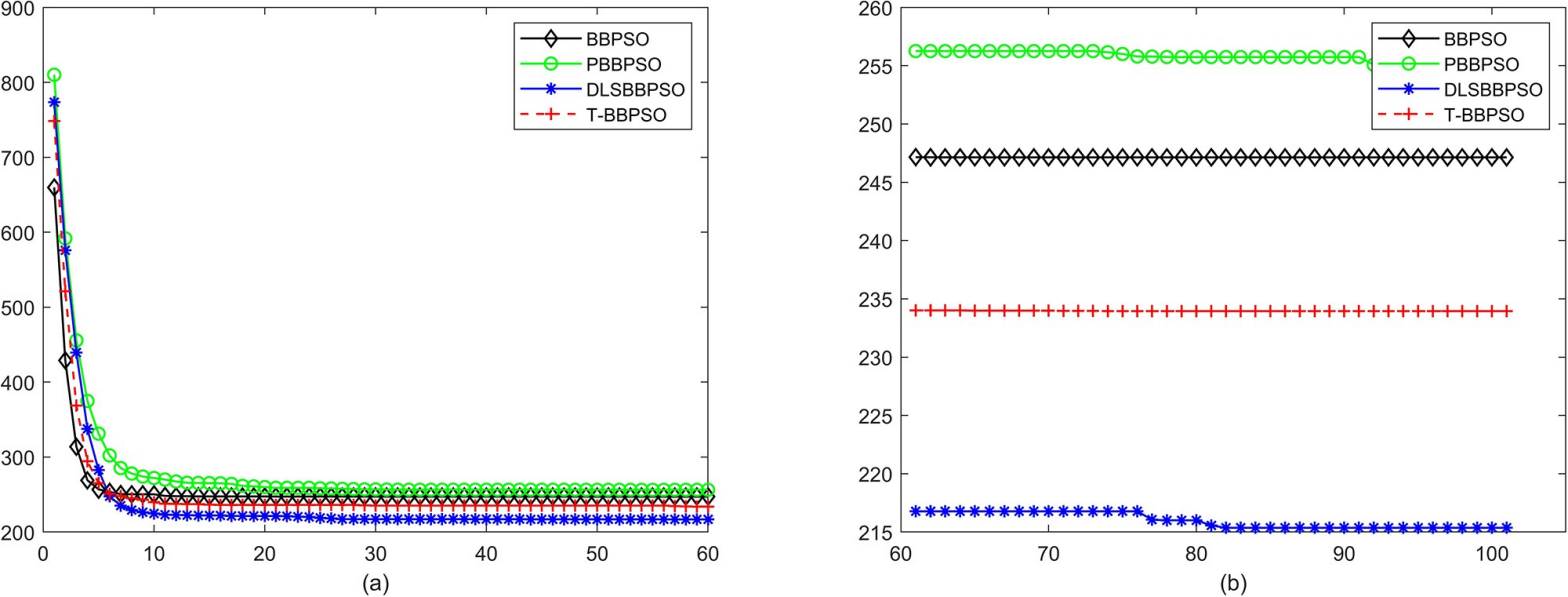
Comparison of convergence speed between BBPSO, PBBPSO, DLS-BBPSO and TBBPSO, *f*_9_, (a) iteration 0–6000, (b) iteration 6000–10000 the unit is 100 iteration.

**Fig 12:**
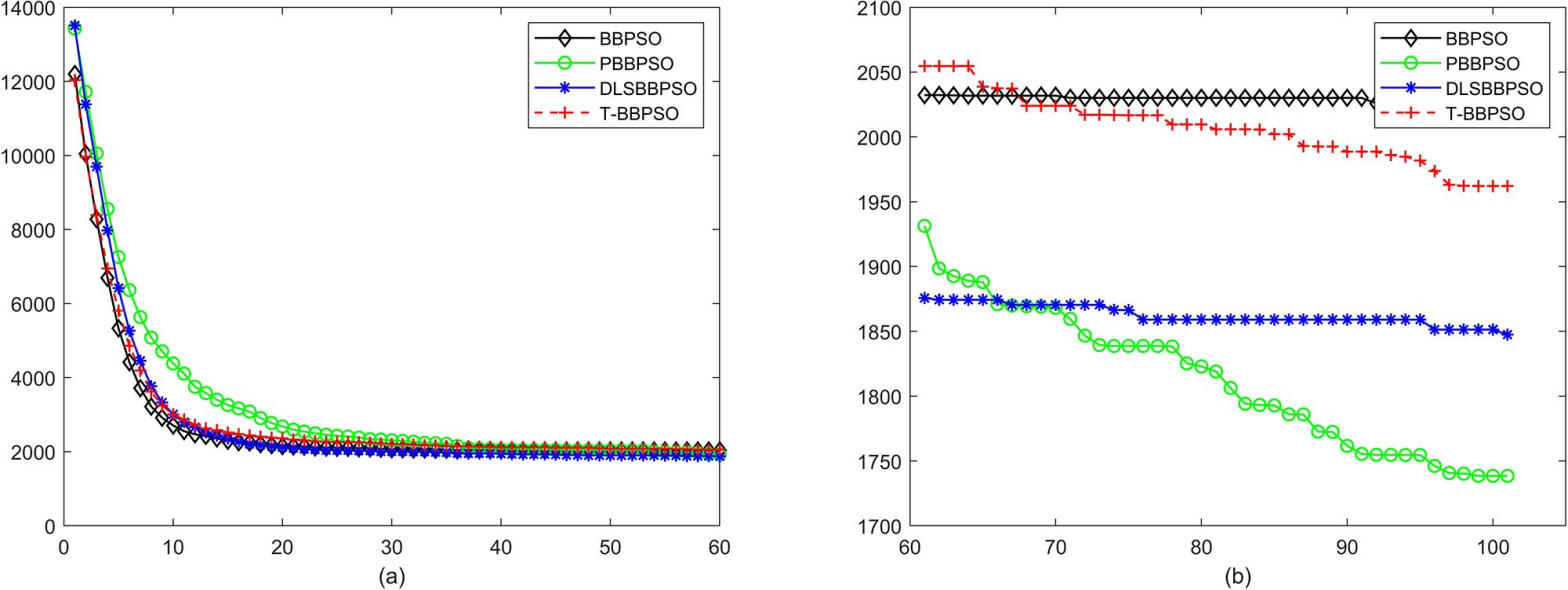
Comparison of convergence speed between BBPSO, PBBPSO, DLS-BBPSO and TBBPSO, *f*_10_, (a) iteration 0–6000, (b) iteration 6000–10000 the unit is 100 iteration.

**Fig 13:**
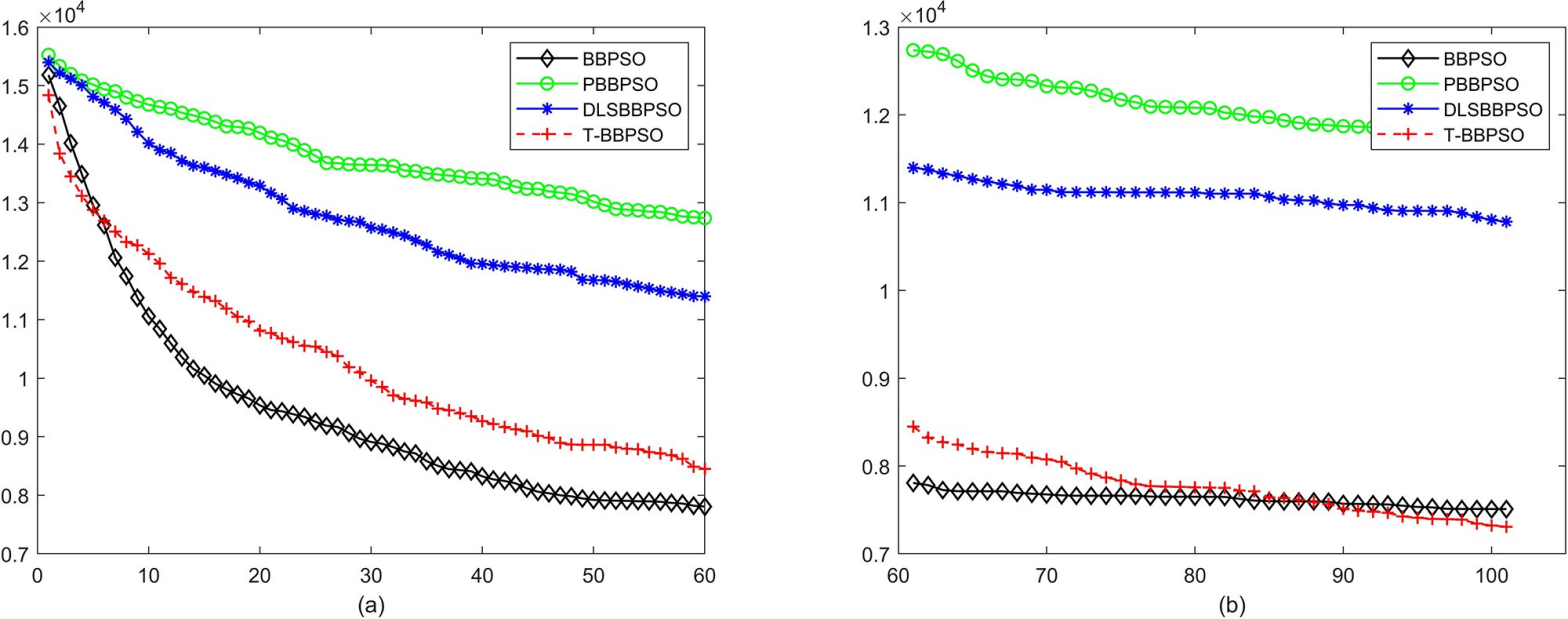
Comparison of convergence speed between BBPSO, PBBPSO, DLS-BBPSO and TBBPSO, *f*_11_, (a) iteration 0–6000, (b) iteration 6000–10000 the unit is 100 iteration.

**Fig 14:**
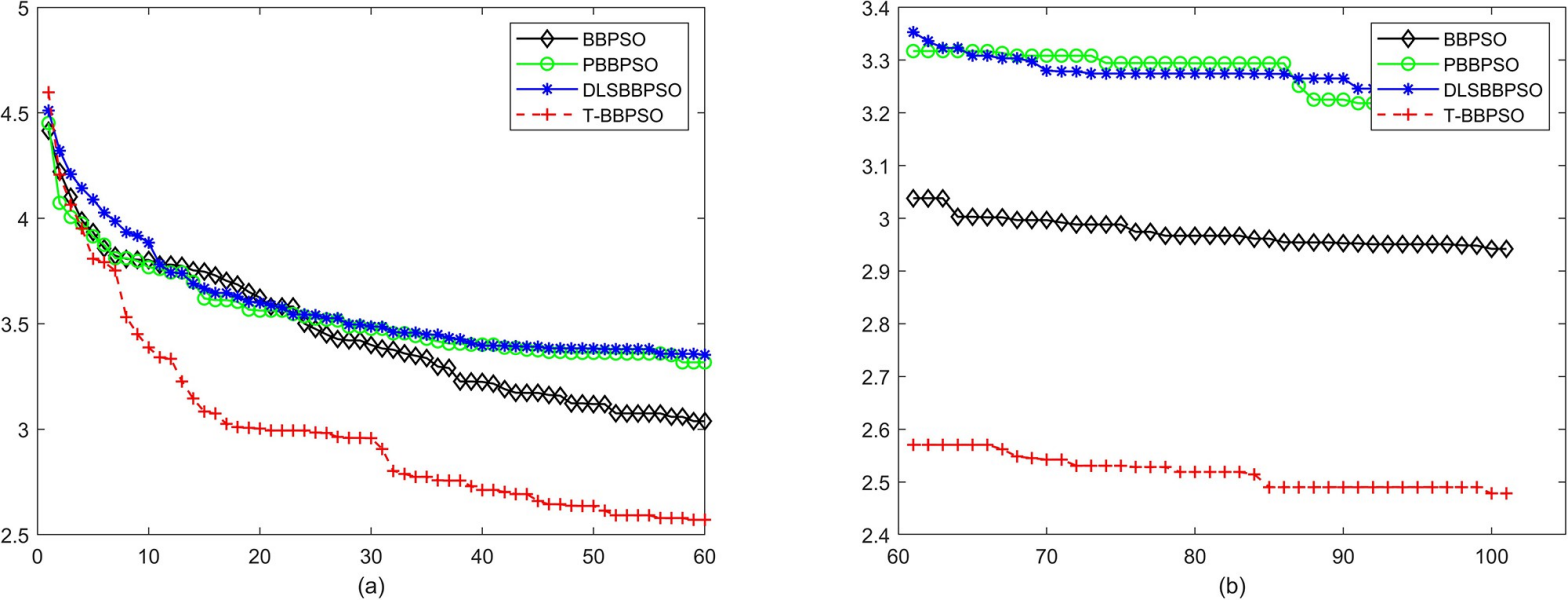
Comparison of convergence speed between BBPSO, PBBPSO, DLS-BBPSO and TBBPSO, *f*_12_, (a) iteration 0–6000, (b) iteration 6000–10000 the unit is 100 iteration.

**Fig 15:**
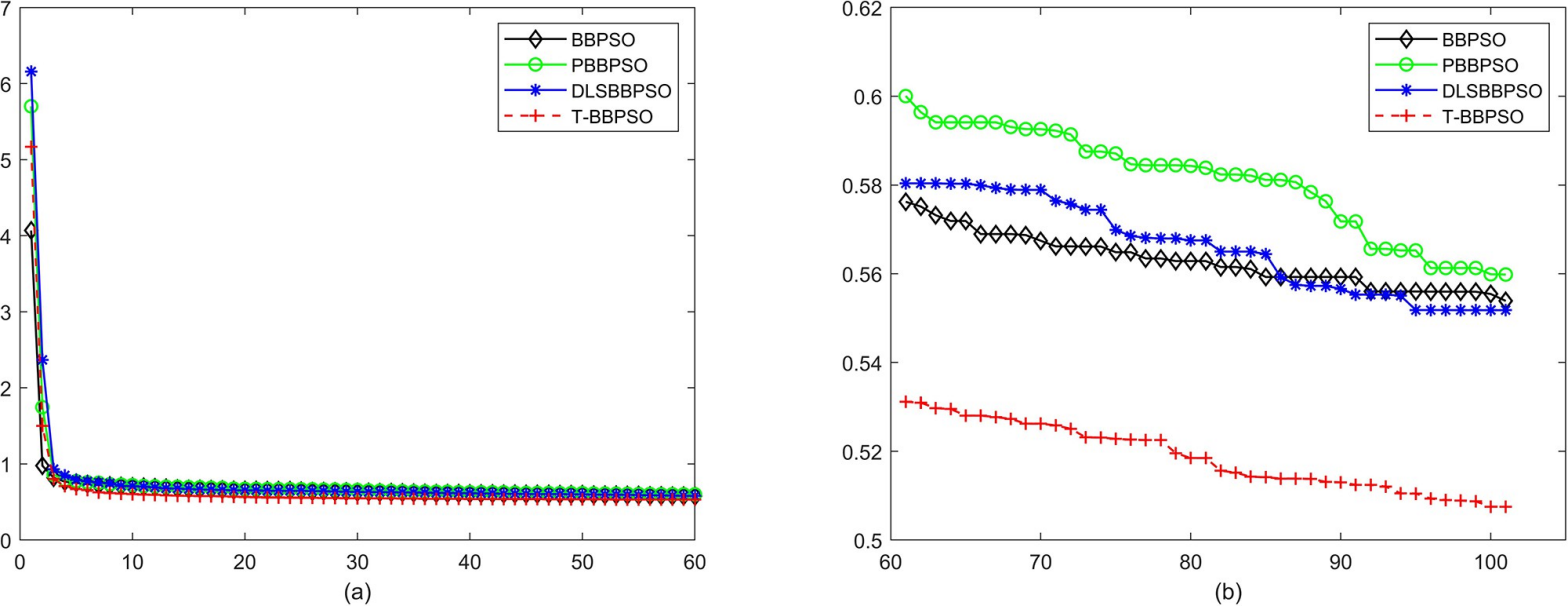
Comparison of convergence speed between BBPSO, PBBPSO, DLS-BBPSO and TBBPSO, *f*_13_, (a) iteration 0–6000, (b) iteration 6000–10000 the unit is 100 iteration.

**Fig 16:**
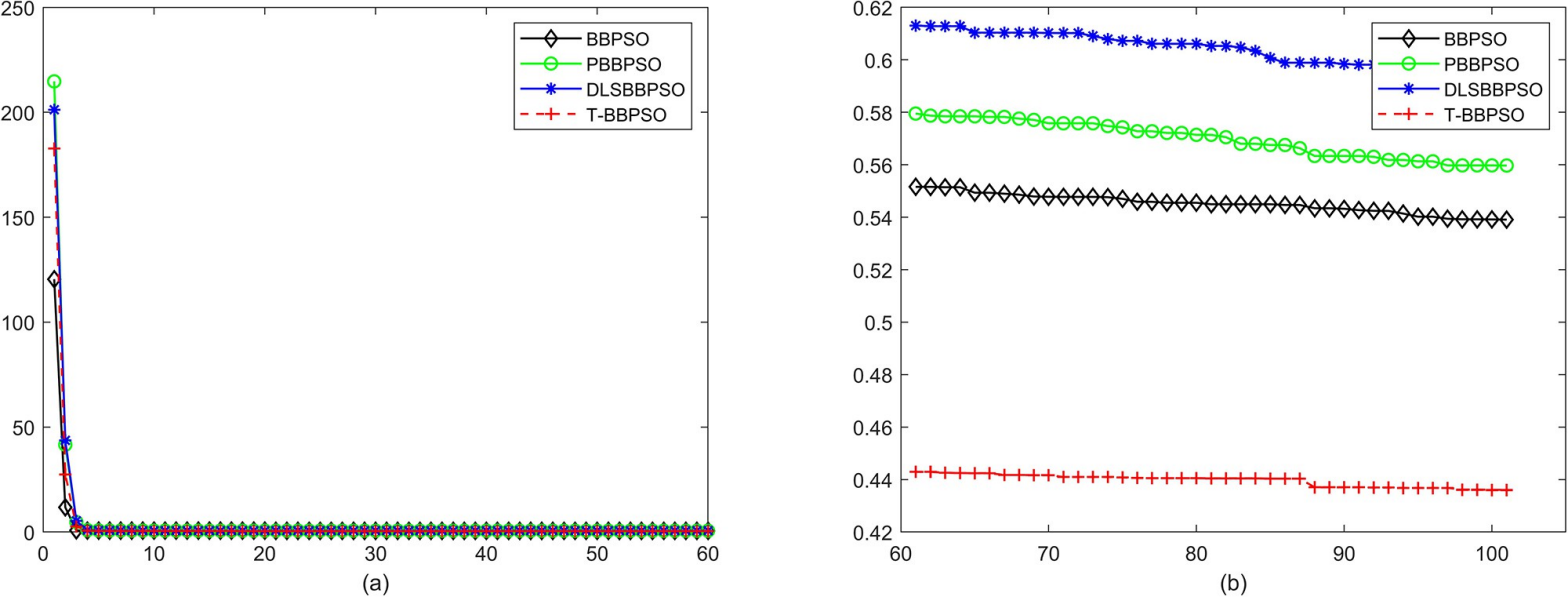
Comparison of convergence speed between BBPSO, PBBPSO, DLS-BBPSO and TBBPSO, *f*_14_, (a) iteration 0–6000, (b) iteration 6000–10000 the unit is 100 iteration.

**Fig 17:**
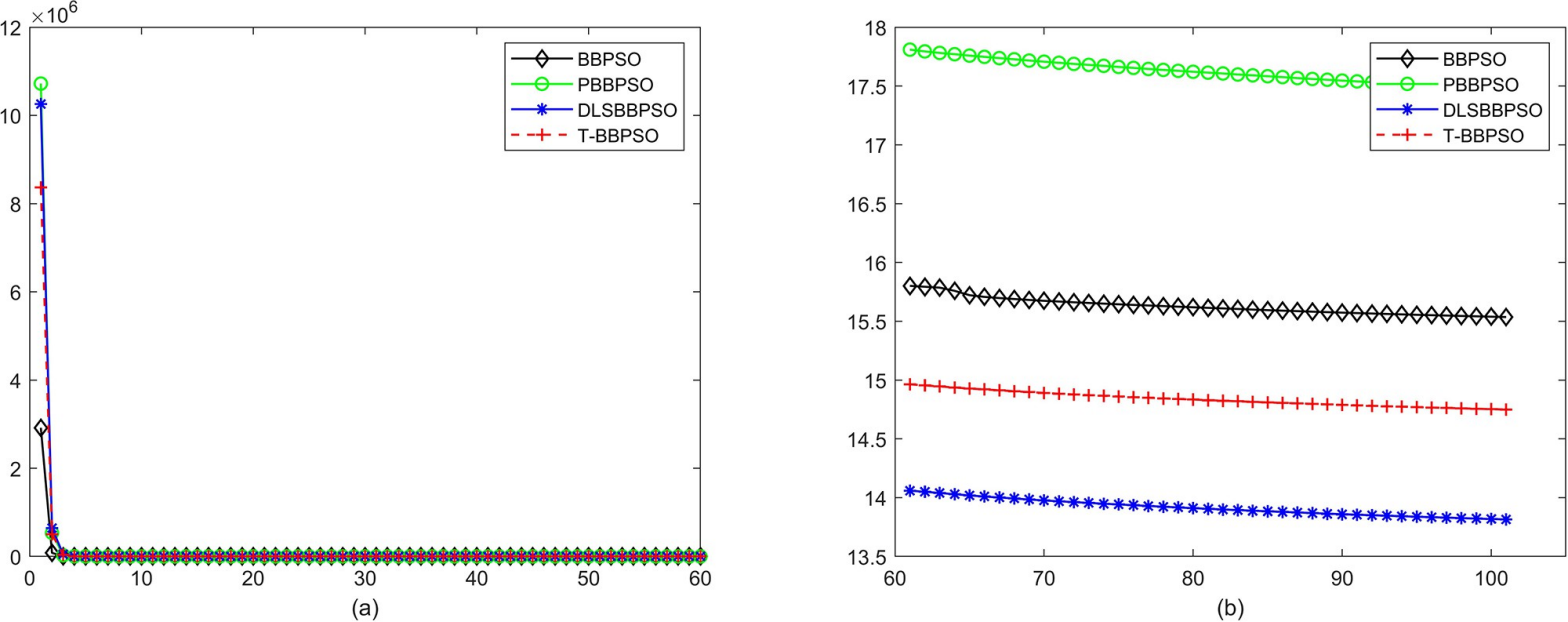
Comparison of convergence speed between BBPSO, PBBPSO, DLS-BBPSO and TBBPSO, *f*_15_, (a) iteration 0–6000, (b) iteration 6000–10000 the unit is 100 iteration.

**Fig 18:**
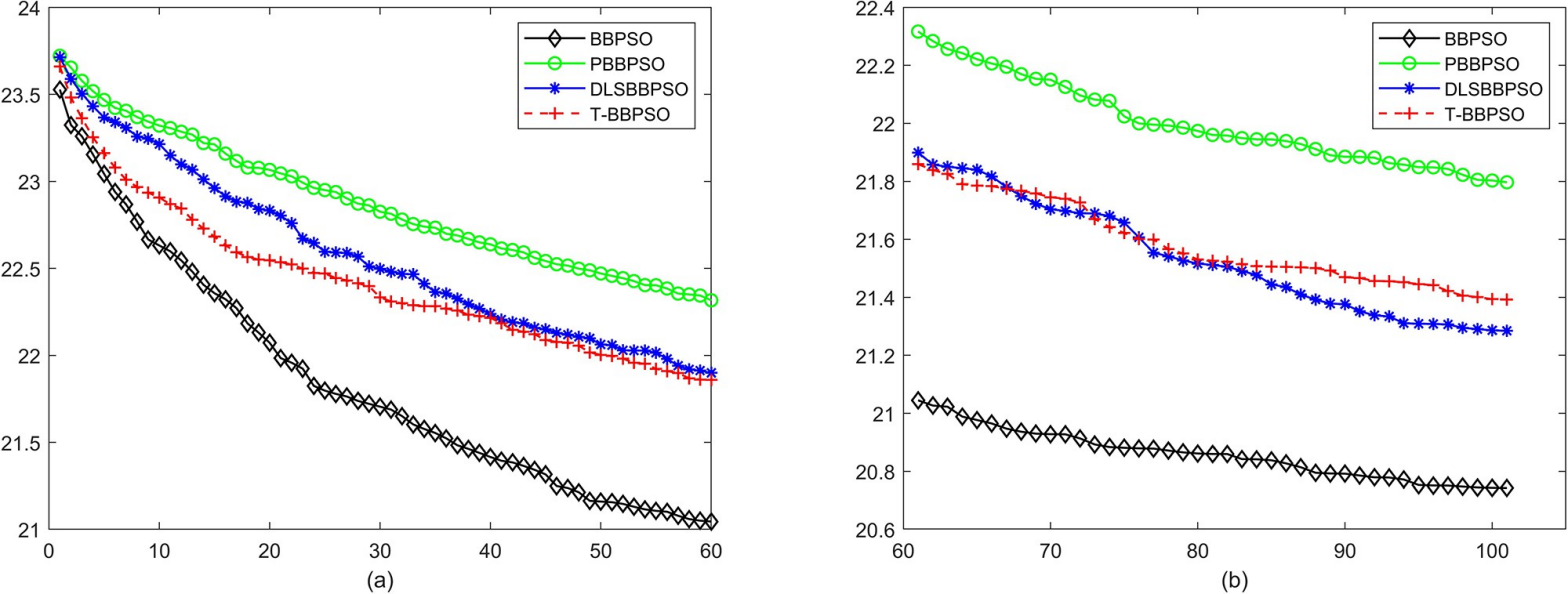
Comparison of convergence speed between BBPSO, PBBPSO, DLS-BBPSO and TBBPSO, *f*_16_, (a) iteration 0–6000, (b) iteration 6000–10000 the unit is 100 iteration.

**Fig 19:**
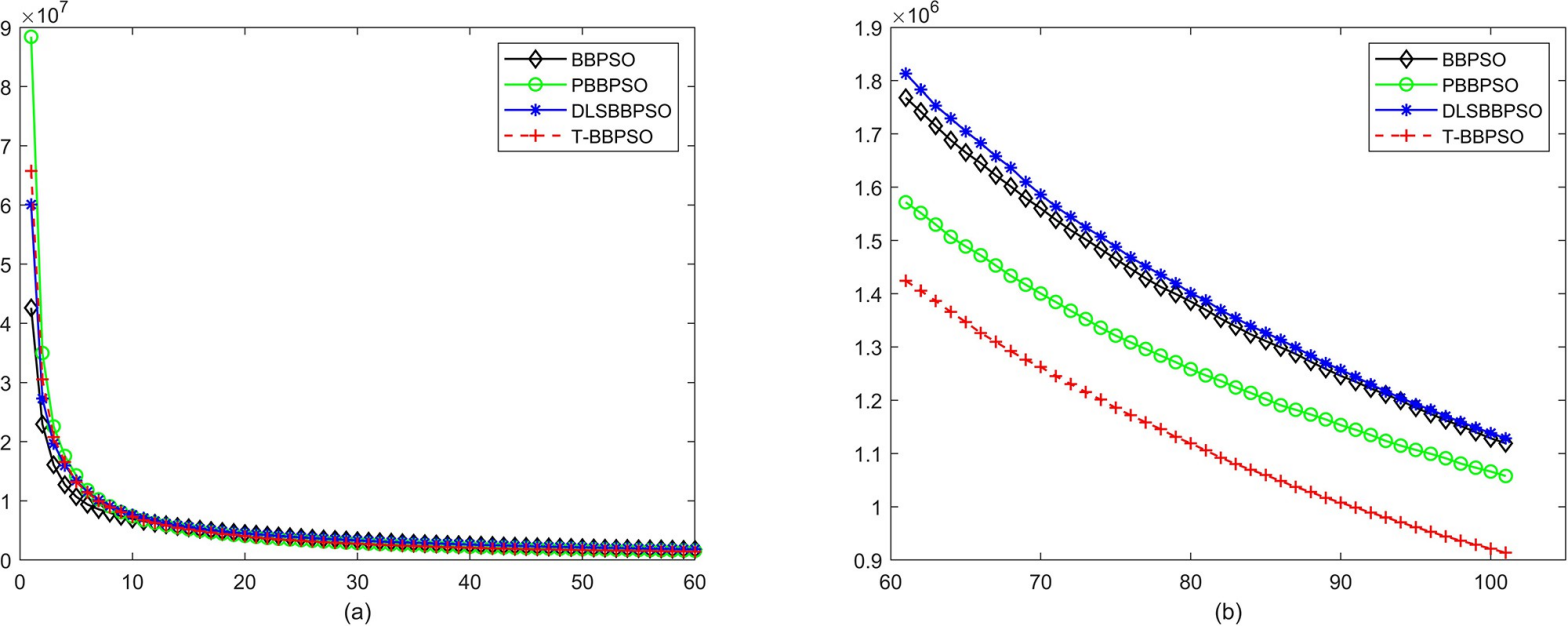
Comparison of convergence speed between BBPSO, PBBPSO, DLS-BBPSO and TBBPSO, *f*_17_, (a) iteration 0–6000, (b) iteration 6000–10000 the unit is 100 iteration.

**Fig 20:**
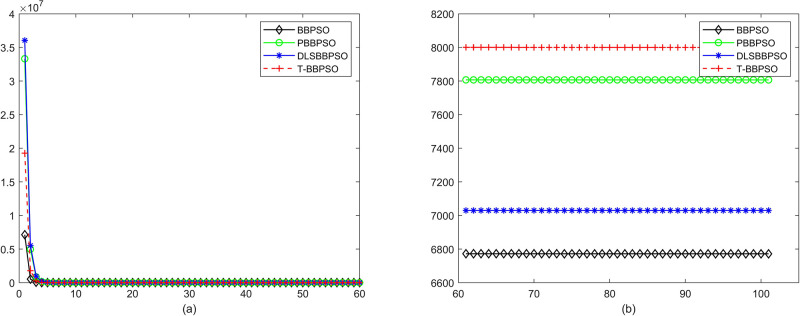
Comparison of convergence speed between BBPSO, PBBPSO, DLS-BBPSO and TBBPSO, *f*_18_, (a) iteration 0–6000, (b) iteration 6000–10000 the unit is 100 iteration.

**Fig 21:**
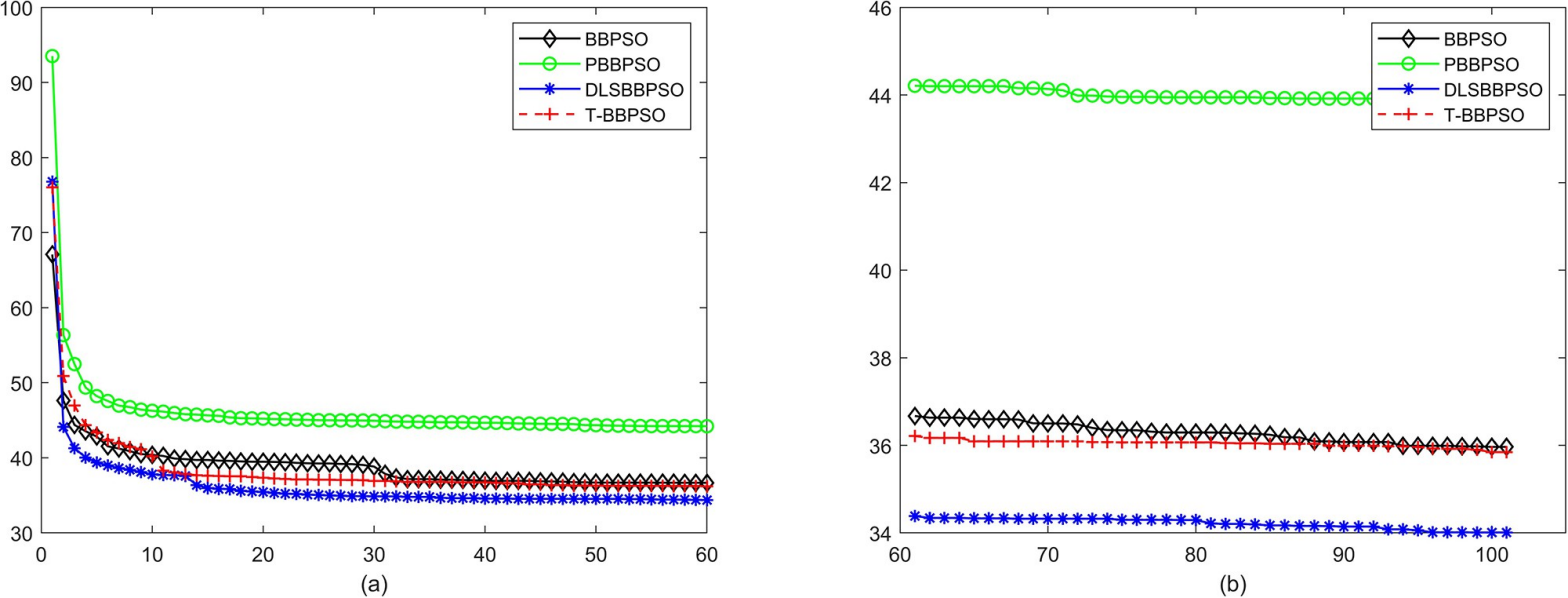
Comparison of convergence speed between BBPSO, PBBPSO, DLS-BBPSO and TBBPSO, *f*_19_, (a) iteration 0–6000, (b) iteration 6000–10000 the unit is 100 iteration.

**Fig 22:**
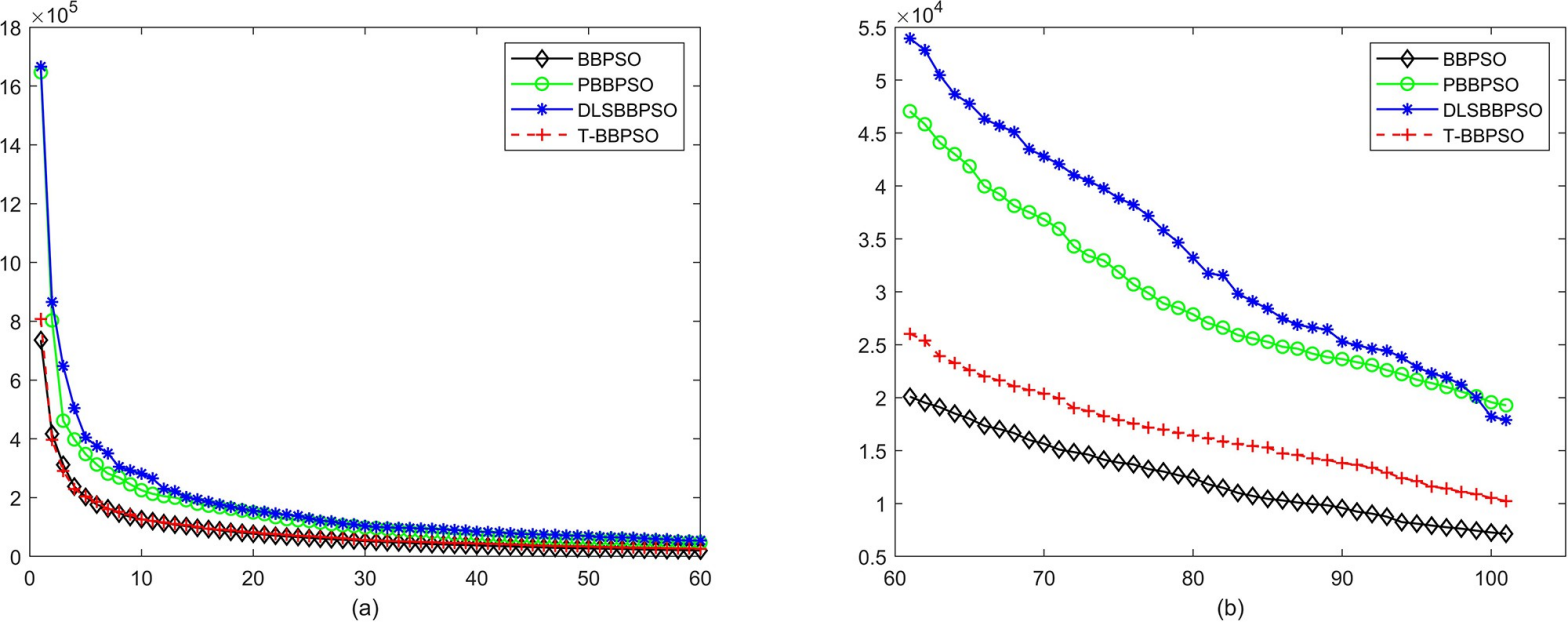
Comparison of convergence speed between BBPSO, PBBPSO, DLS-BBPSO and TBBPSO, *f*_20_, (a) iteration 0–6000, (b) iteration 6000–10000 the unit is 100 iteration.

**Fig 23:**
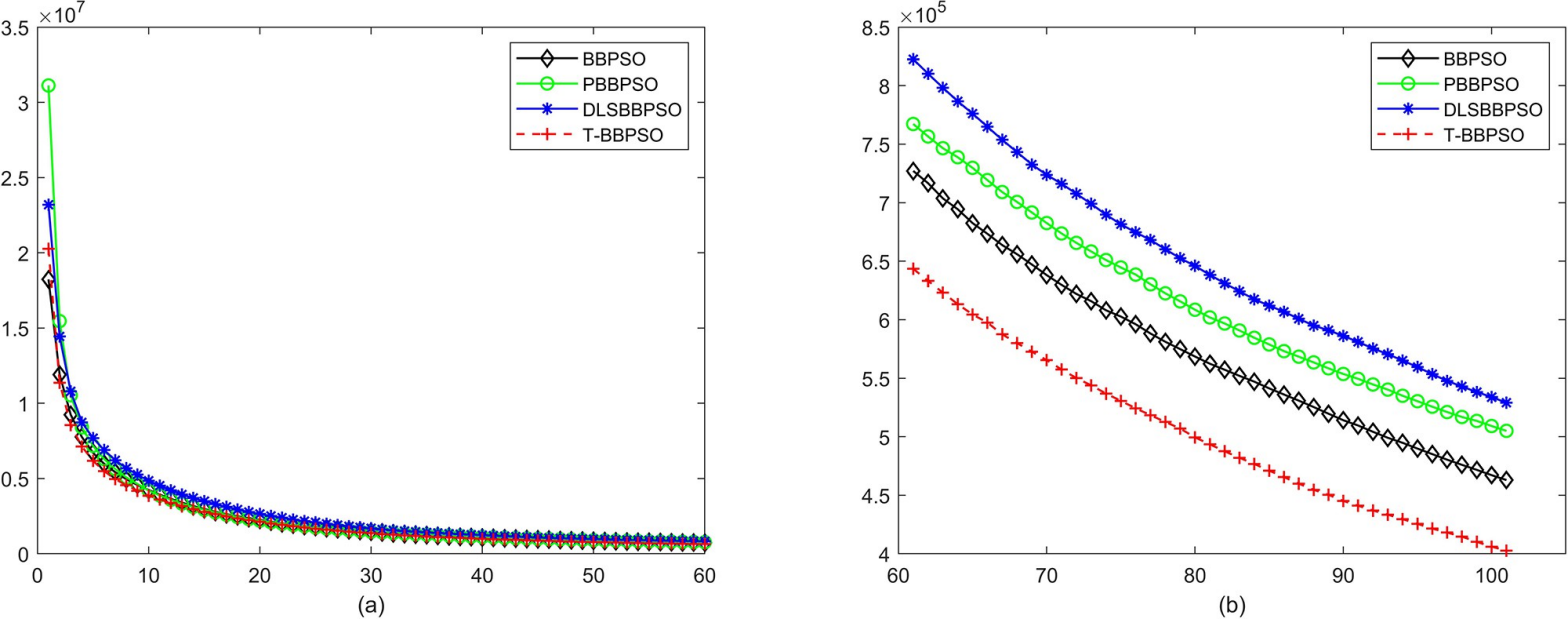
Comparison of convergence speed between BBPSO, PBBPSO, DLS-BBPSO and TBBPSO, *f*_21_, (a) iteration 0–6000, (b) iteration 6000–10000 the unit is 100 iteration.

**Fig 24:**
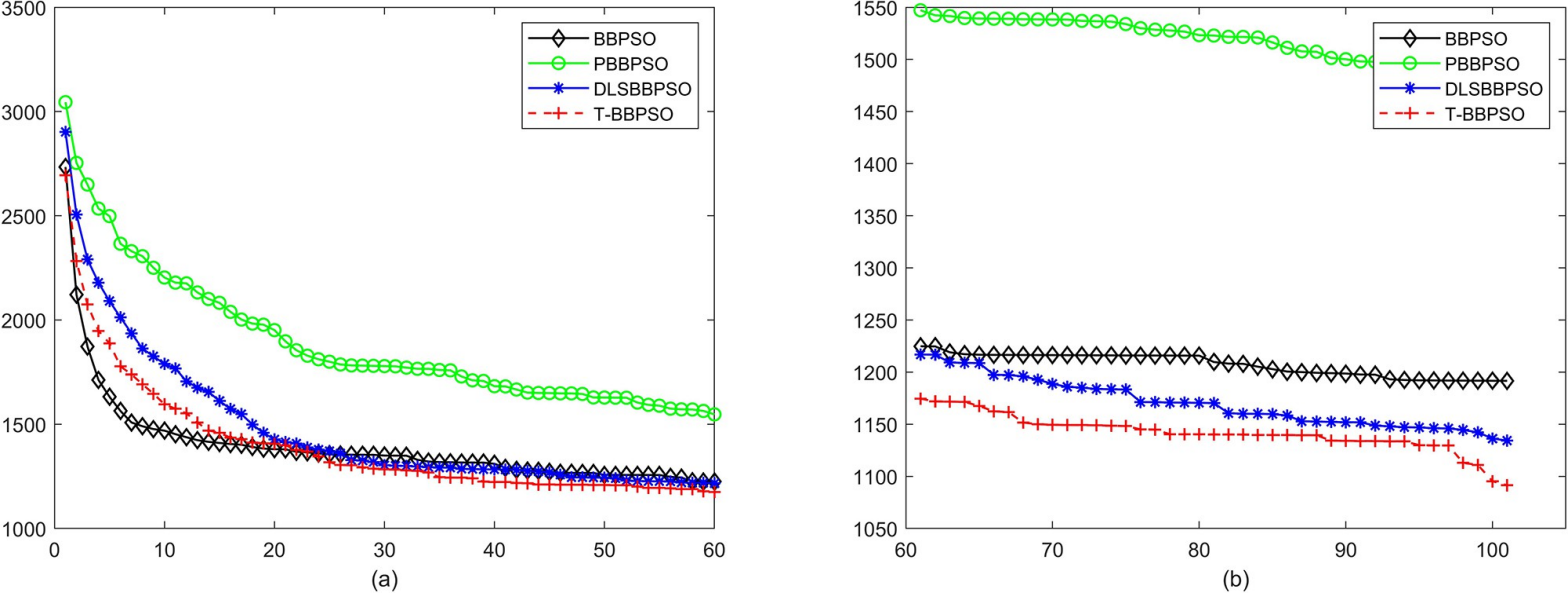
Comparison of convergence speed between BBPSO, PBBPSO, DLS-BBPSO and TBBPSO, *f*_22_, (a) iteration 0–6000, (b) iteration 6000–10000 the unit is 100 iteration.

**Fig 25:**
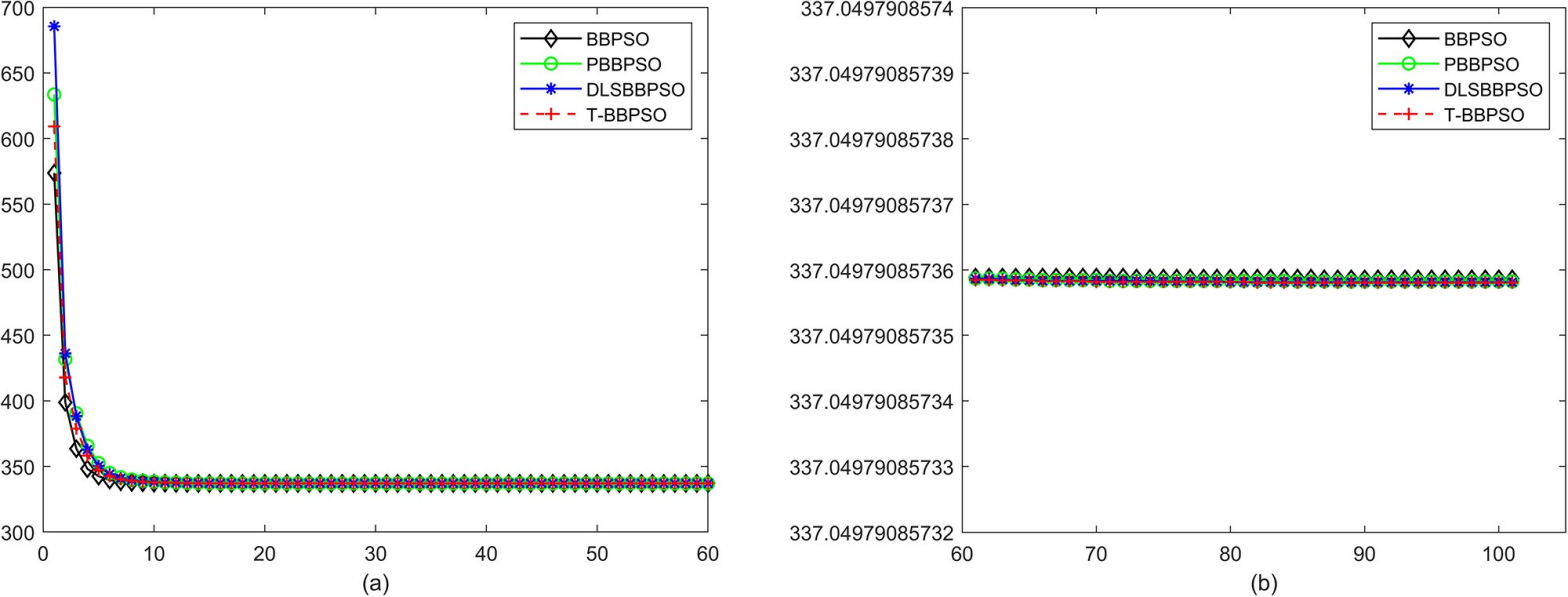
Comparison of convergence speed between BBPSO, PBBPSO, DLS-BBPSO and TBBPSO, *f*_23_, (a) iteration 0–6000, (b) iteration 6000–10000 the unit is 100 iteration.

**Fig 26:**
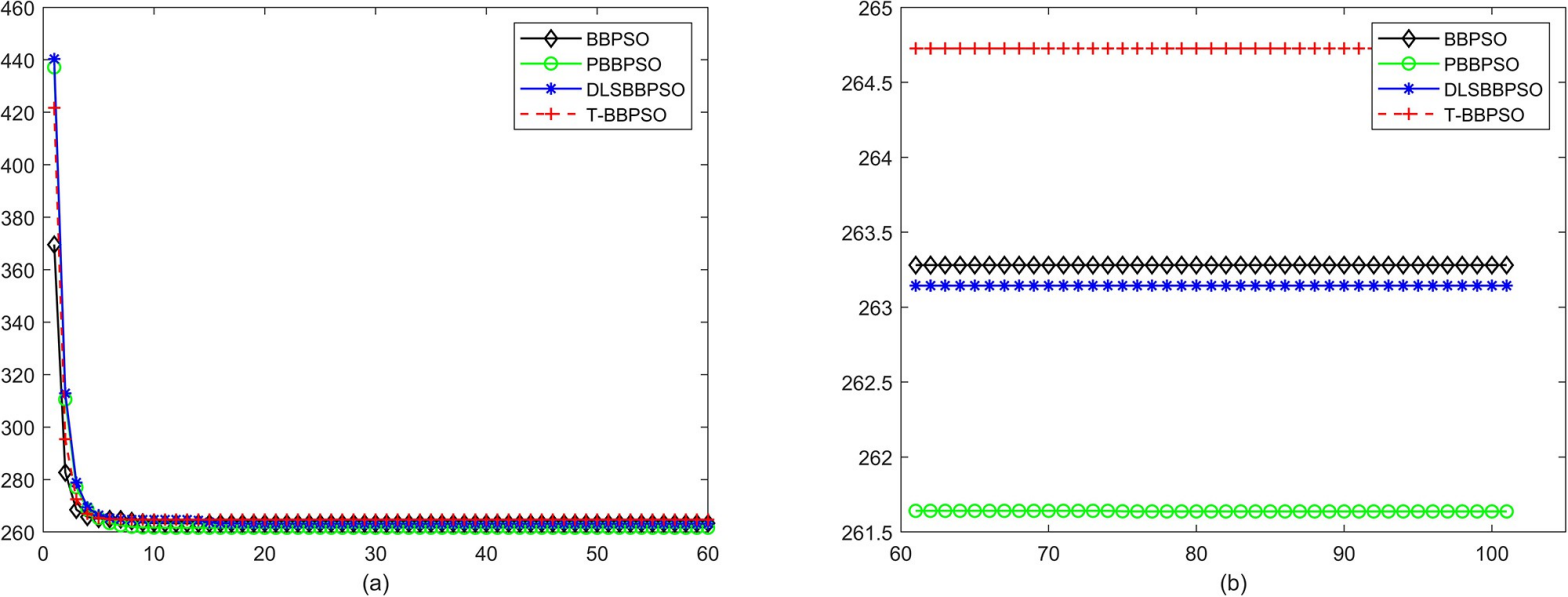
Comparison of convergence speed between BBPSO, PBBPSO, DLS-BBPSO and TBBPSO, *f*_24_, (a) iteration 0–6000, (b) iteration 6000–10000 the unit is 100 iteration.

**Fig 27:**
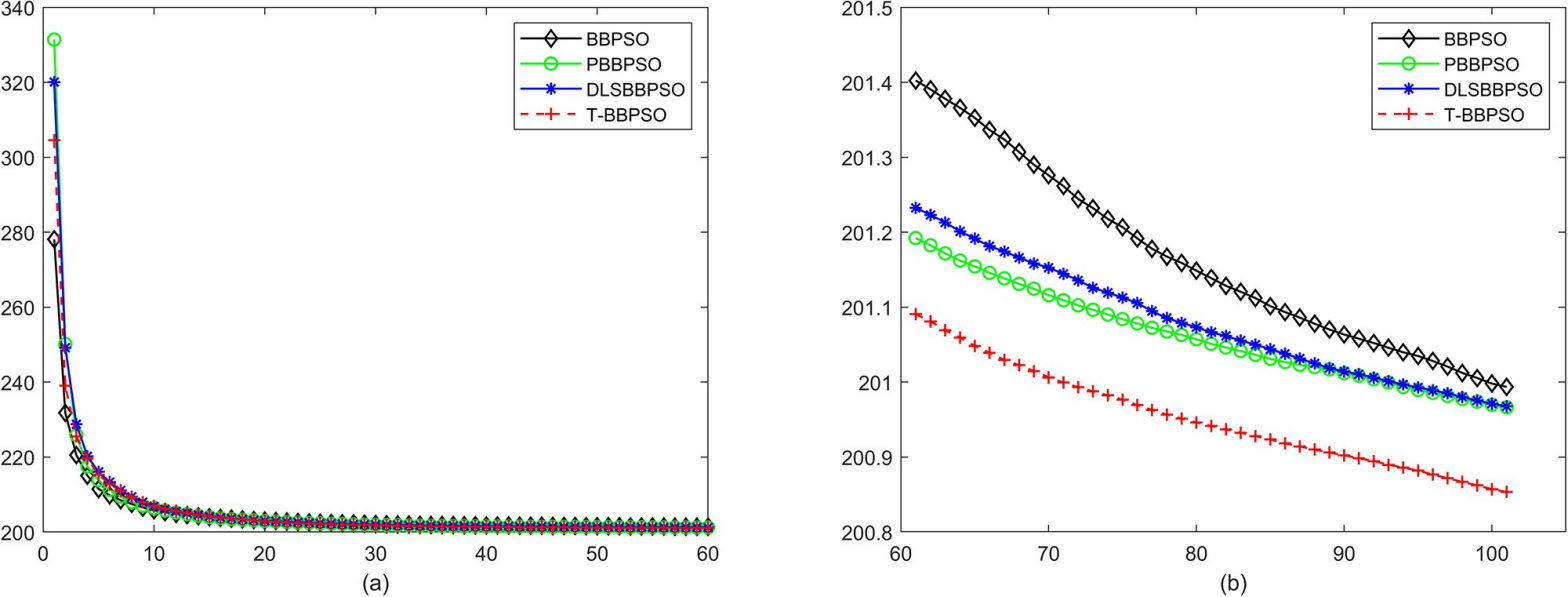
Comparison of convergence speed between BBPSO, PBBPSO, DLS-BBPSO and TBBPSO, *f*_25_, (a) iteration 0–6000, (b) iteration 6000–10000 the unit is 100 iteration.

**Fig 28:**
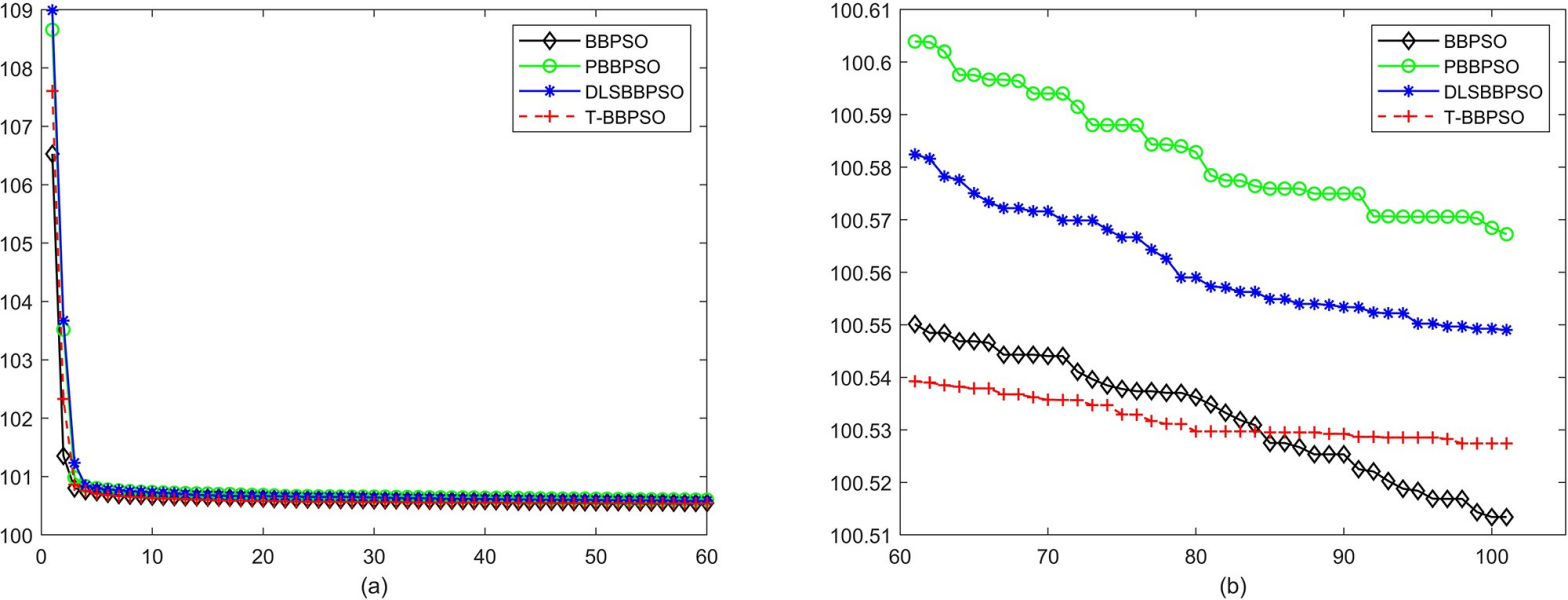
Comparison of convergence speed between BBPSO, PBBPSO, DLS-BBPSO and TBBPSO, *f*_26_, (a) iteration 0–6000, (b) iteration 6000–10000 the unit is 100 iteration.

**Fig 29:**
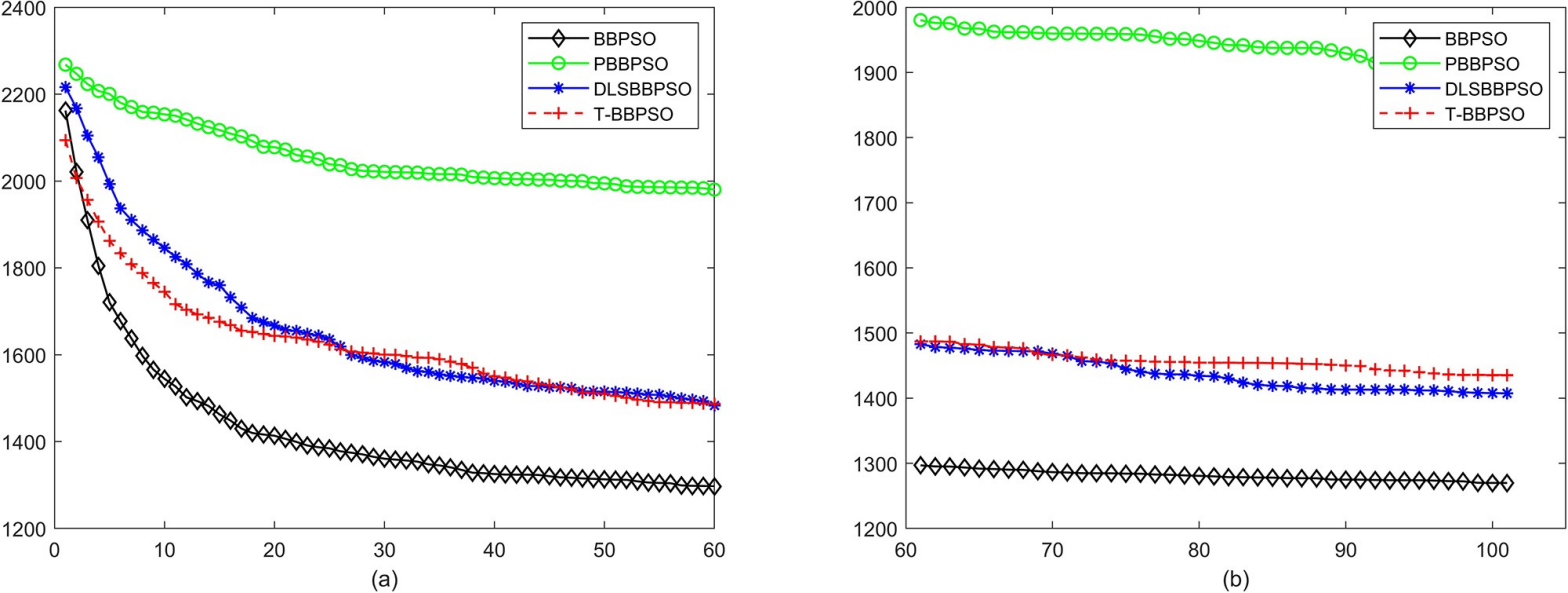
Comparison of convergence speed between BBPSO, PBBPSO, DLS-BBPSO and TBBPSO, *f*_27_, (a) iteration 0–6000, (b) iteration 6000–10000 the unit is 100 iteration.

**Fig 30:**
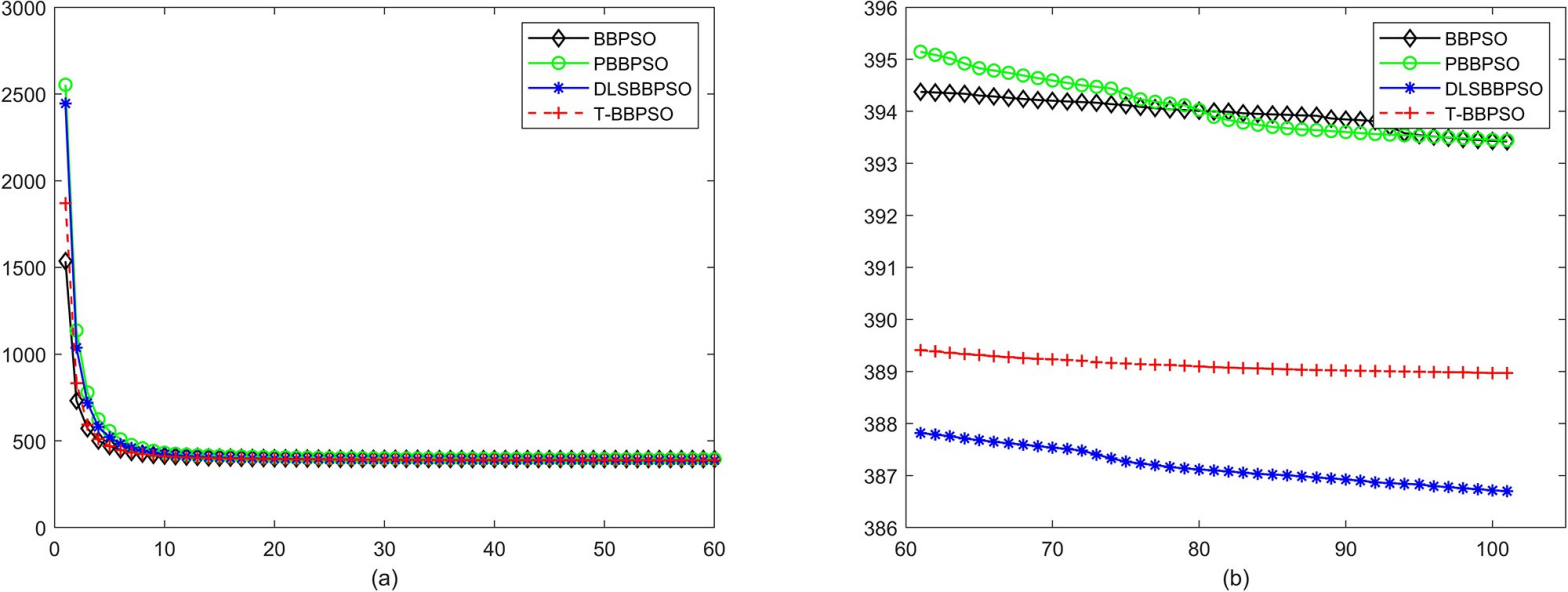
Comparison of convergence speed between BBPSO, PBBPSO, DLS-BBPSO and TBBPSO, *f*_28_, (a) iteration 0–6000, (b) iteration 6000–10000 the unit is 100 iteration.

**Fig 31:**
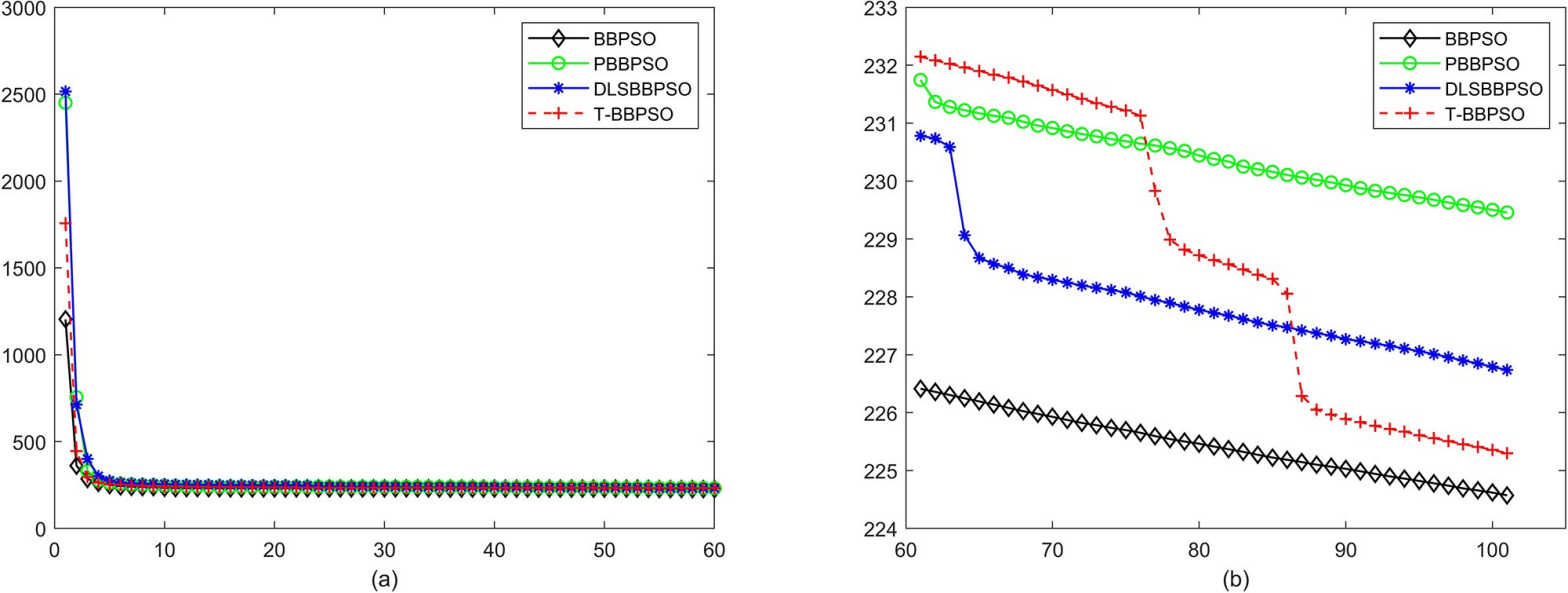
Comparison of convergence speed between BBPSO, PBBPSO, DLS-BBPSO and TBBPSO, *f*_29_, (a) iteration 0–6000, (b) iteration 6000–10000 the unit is 100 iteration.

**Fig 32:**
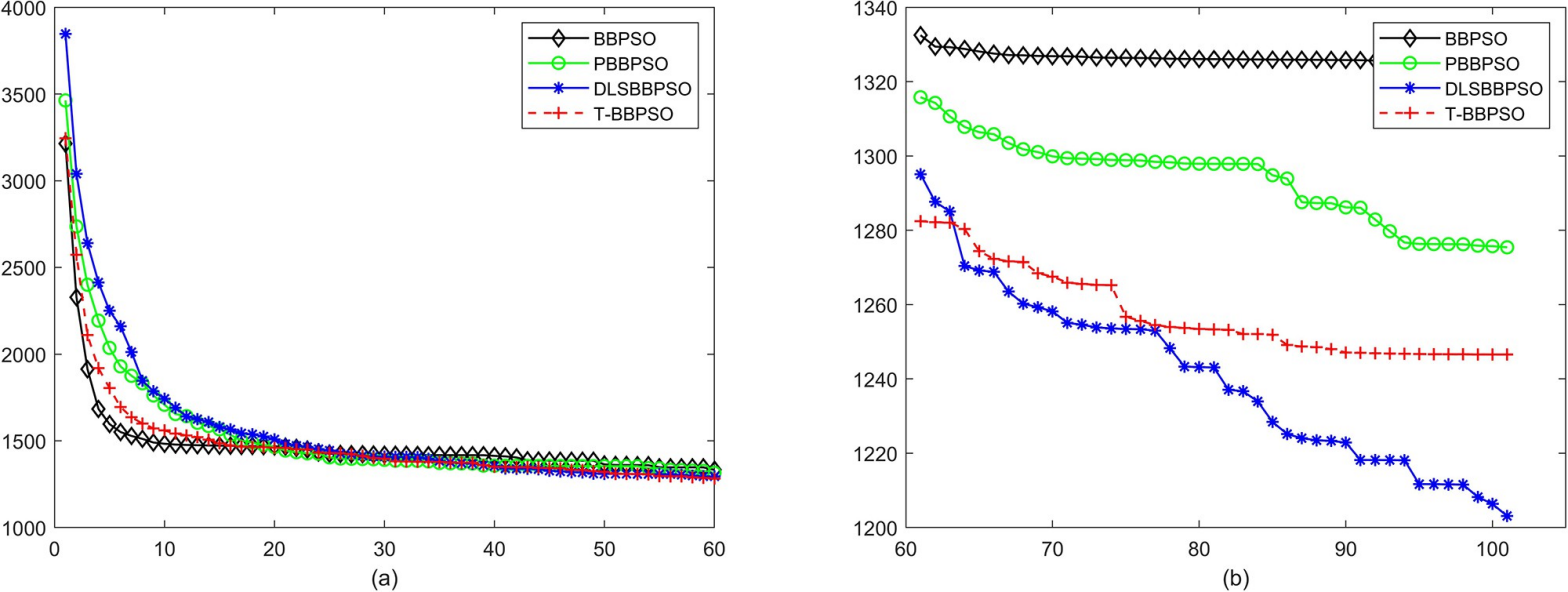
Comparison of convergence speed between BBPSO, PBBPSO, DLS-BBPSO and TBBPSO, *f*_30_, (a) iteration 0–6000, (b) iteration 6000–10000 the unit is 100 iteration.

### Discussion and future works

The main reason that TBBPSO can provide high precision results for test functions is that the TGO divides the original swarm into several local groups. This process increases the diversity of the swarm and enhances exploration. The MO makes the main local group merge one local group per iteration while the rest local groups can keep doing a local search. These processes increase the exploitation.

The overall effectiveness (OE) [21] of TBBPSO and other algorithms in the control group is computed by results in Tables 3 and 4. The OE is calculated by Eq 4.

$$\begin{array}{c} \begin{array}{c} OE=\frac{N-L}{N}*100\% \end{array} \end{array}$$ (4)

where *N* is the number of test functions, *L* is the number of times the target algorithm loses in the competition. Results pf OE is shown in Table 5. Four algorithms tie in *f*_23_. It can be seen that TBBPSO has the best performance.

**Table 5:** OE Results of BBPSO, PBBPSO, DLS-BBPSO and TBBPSO.

Dimension	BBPSO [17]	PBBPSO [34]	DLS-BBPSO [35]	TBBPSO
OE	33.33$	13.33%	23.33%	40.00%

In the last iteration, all the particles are concentrated at one point, so it is impossible to find a solution with a better position. How to maintain the diversity of the particle population even after very long iterations will be the main direction of future work. The TGO-MO corporation pattern works well in single-objective optimizations. Another direction of the future work is to apply this work pattern to multi-objective optimization problems.

## Conclusions

A TBBPSO is proposed in this paper to solve single-objective optimization problems. The TBBPSO works by re-organizing the structure of the particle swarm. The TGO and MO cooperate to prevent the particle swarm from losing diversity too fast. The TBBPSO presents high-precision results when handling CEC 2014 benchmark functions. The main reason is the cooperation of the TGO and the MO. The TGO divides the particle swarm into several calculation units, the twins. Each twin contains two particles playing different roles. The particle with a better personal best position plays as the team leader, the other one plays as the team member. Different evolution strategies apply to different roles. Then one twin will be selected as the main local group (MLG) and the other twins will be sub-local groups (SLGs). This strategy can increase the diversity of the particle swarm and give the swarm more chance to escape from local minimums. In the MO, the MLG will merge one SLG in every iteration. With the increase of the population, the local search capability of the MLG is increased. At the same time, other SLGs will search independently of each other to ensure the swarm keeps the ability to escape from local minimums. When all SLGs are merged by the MLS, all particles are in the same group and the algorithm goes to the TGO. This process is simple and fast, no parameter, congestion, or threshold is needed, and the time complexity is *o*(*n*).

## Supporting information

S1 File Experimental results. (MAT)

S2 File Original code of TBBPSO. (M)
